# The efficacy of real versus sham external Trigeminal Nerve Stimulation (eTNS) in youth with Attention-Deficit/Hyperactivity Disorder (ADHD) over 4 weeks: a protocol for a multi-centre, double-blind, randomized, parallel-group, phase IIb study (ATTENS)

**DOI:** 10.1186/s12888-024-05650-1

**Published:** 2024-04-30

**Authors:** Katya Rubia, Lena Johansson, Ben Carter, Dominic Stringer, Paramala Santosh, Mitul A. Mehta, Aldo Alberto Conti, Natali Bozhilova, Irem Ece Eraydin, Samuele Cortese

**Affiliations:** 1 Department of Child & Adolescent Psychiatry/PO46 Institute of Psychiatry, Psychology & Neurosciences King’s College London, De Crespigny Park, London, SE5 8AF UK; 2 Department of Child & Adolescent Psychiatry, Technical University, Dresden, Germany; 3 Department of Biostatistics and Health Informatics, Institute of Psychiatry, Psychology & Neuroscience, King’s College London, London, UK; 4 King’s Clinical Trial Unit, Institute of Psychiatry, Psychology & Neuroscience, King’s College London, London, UK; 5 National and Specialist CAMHS, South London and Maudsley NHS Foundation Trust, London, UK; 6 Department for Neuroimaging, Institute of Psychiatry, Psychology & Neuroscience, King’s College London, London, UK; 7 Centre for Innovation in Mental Health, School of Psychology, University of Southampton, Southampton, UK; 8 Clinical and Experimental Sciences (CNS and Psychiatry), Faculty of Medicine, University of Southampton, Southampton, UK; 9 SOLENT NHS Trust, Southampton, UK; 10 Hassenfeld Children’s Hospital at NYU Langone, New York University Child Center, New York City, NY USA

**Keywords:** Attention-Deficit, Hyperactivity Disorder, ADHD, External trigeminal nerve stimulation, Trigeminal nerve stimulation, eTNS, TNS, Transcutaneous electrical nerve stimulation, TENS, External transcutaneous Trigeminal Nerve Stimulation, Supraorbital transcutaneous stimulation, Transcutaneous external supraorbital nerve stimulation, Transcutaneous supraorbital nerve stimulation, Trigeminal transcutaneous nerve stimulation, Functional magnetic resonance imaging, fMRI, Executive functions, EF

## Abstract

**Background:**

Attention Deficit/Hyperactivity Disorder (ADHD), if severe, is usually treated with stimulant or non-stimulant medication. However, users prefer non-drug treatments due to side effects. Alternative non-medication treatments have so far only shown modest effects. External trigeminal nerve stimulation (eTNS) is a minimal risk, non-invasive neuromodulation device, targeting the trigeminal system. It was approved for ADHD in 2019 by the USA Food and Drug administration (FDA) based on a small proof of concept randomised controlled trial (RCT) in 62 children with ADHD showing improvement of ADHD symptoms after 4 weeks of nightly real versus sham eTNS with minimal side effects. We present here the protocol of a larger confirmatory phase IIb study testing efficacy, longer-term persistency of effects and underlying mechanisms of action.

**Methods:**

A confirmatory, sham-controlled, double-blind, parallel-arm, multi-centre phase IIb RCT of 4 weeks of eTNS in 150 youth with ADHD, recruited in London, Portsmouth, and Southampton, UK. Youth with ADHD will be randomized to either real or sham eTNS, applied nightly for 4 weeks. Primary outcome is the change in the investigator-administered parent rated ADHD rating scale. Secondary outcomes are other clinical and cognitive measures, objective hyperactivity and pupillometry measures, side effects, and maintenance of effects over 6 months. The mechanisms of action will be tested in a subgroup of 56 participants using magnetic resonance imaging (MRI) before and after the 4-week treatment.

**Discussion:**

This multi-centre phase IIb RCT will confirm whether eTNS is effective in a larger age range of children and adolescents with ADHD, whether it improves cognition and other clinical measures, whether efficacy persists at 6 months and it will test underlying brain mechanisms. The results will establish whether eTNS is effective and safe as a novel non-pharmacological treatment for ADHD.

**Trial registration**: ISRCTN82129325 on 02/08/2021, https://doi.org/10.1186/ISRCTN82129325.

**Supplementary Information:**

The online version contains supplementary material available at 10.1186/s12888-024-05650-1.

## Background and rationale

Attention-deficit/hyperactivity disorder (ADHD) affects around 5–7% of children and is defined by the DSM-5 as age-inappropriate and impairing symptoms of inattention and/or impulsiveness/hyperactivity (DSM 5) [1]. Symptoms persist into adulthood in most cases, and they are associated with worse academic and social outcomes. Children with ADHD may have impairments in one or more executive functions, most prominently in sustained attention, working memory, and inhibitory control. These impairments are associated with abnormal function of the mediating fronto-striatal, fronto-cerebellar and fronto-parietal regions and networks [2–5]. Stimulant medication is the mainstream treatment, but it may not be preferred by users due to commonly occurring side effects, is not suitable for all patients, and compliance may be poor especially in adolescence [6, 7]. Alternative non-pharmacological treatments with fewer side effects are desirable but have so far only shown modest effects [5]. External Trigeminal Nerve Stimulation (eTNS) is the first non-pharmacological treatment approved by the USA Food and Drug administration (FDA) for ADHD. eTNS is a minimal risk, non-invasive neuromodulation device that sends low electrical pulses under the skin on the forehead targeting the trigeminal system and can be applied during sleep. TNS transmits small electrical currents under the skin of the forehead through a self-adhesive electrode, over the supraorbital area that triggers action potentials to the supraorbital and supratrochlear branches of the ophthalmic nerve, which is a branch of the first trigeminal division (V1). The trigeminal nerve has several connections to the brain, in particular to locus coeruleus (LC), the reticular activation system, the brain stem, as well as frontal, other cortical and thalamic areas. Furthermore, it has effects on several neurotransmitters, in particular noradrenaline and dopamine. These brain areas and catecholamines are related to attention and arousal [8] and have been shown to be dysfunctional in ADHD [2, 4, 5].

The FDA approval of eTNS was based on data from a proof-of-concept randomised controlled trial (RCT) in 62 children with ADHD between 8–12 years of age, showing improvement of ADHD symptoms after 4 weeks of real compared to sham eTNS administered every night for 8 h by parents [9]. Specifically, the main outcome measure, the investigator-scored, parent-rated ADHD-RS total score, was significantly reduced in the active relative to the sham group after 4 weeks of treatment with a medium effect size (Cohen’s d = 0.5). The inattentive and hyperactive/impulsive sub-scores and the Clinical Global Impression-Improvement scores were also improved in active versus sham treatment. There was furthermore a trend-level differential improvement in the active versus sham group for anxiety but not for depression [9]. There were relatively minor and transient side effects such as headaches or fatigue, with no serious adverse events reported. Quantitative electroencephalography (qEEG) data showed increased power in right inferior and midline frontal regions in the active relative to the sham group which were correlated with ADHD-RS total score and the ADHD hyperactive-impulsive subscores improvements which indicates that they may mediate the clinical improvements [9]. These qEEG findings are parallel to animal and human neuroimaging studies that demonstrate that eTNS activates prefrontal brain regions, as well as subcortical regions such as thalamus, insula and anterior cingulate, the reticular activation system and the locus coeruleus, [8, 10, 11]. An earlier, open trial from the same group of eTNS of 6 weeks in children with ADHD showed additional improvements of executive functions concomitant with clinical improvement [12].

Based on evidence from this small proof of concept sham-controlled randomised controlled trial, eTNS is now FDA approved for ADHD. However, more evidence is clearly needed to demonstrate the efficacy and effectiveness of eTNS for reducing ADHD symptoms and to understand its currently relatively unknown underlying mechanisms of action. There is also a need to test its efficacy in older children and adolescents where compliance with medication is typically poor [13].

Here, we present the protocol of a study whose objective is to conduct a well-powered confirmatory, randomised sham controlled RCT of eTNS across two sites, London, and Southampton, with recruitment across three cities (London, Southampton, and Portsmouth) over 4 weeks in 150 youth with ADHD to establish/confirm whether eTNS is efficacious in improving ADHD symptoms and cognitive functions and to test potential longer-term effects after 6 months. We will measure the same primary and some secondary clinical outcome measures as the USA trial as well as additional ADHD-relevant clinical measures such as mind-wandering, which is a key feature of ADHD, and sleep quality. We will also measure objective hyperactivity indices based on a wrist-hand device. Furthermore, we will assess a wider range of ADHD-relevant executive functions than the previous trials (which only measured attention and working memory) to assess effects of eTNS more thoroughly on cognition. Our task battery includes additional measures of motor and interference inhibition, sustained attention, vigilance, and timing, providing a more comprehensive assessment of potential improvements in cognition. All measures will be repeated at 6 months to test for longer-term effects. Also, to understand the underlying mechanisms of action, the previous trial used EEG, which has poor spatial resolution and is not ideal for a treatment like eTNS, which is believed to work predominantly subcortically which cannot be assessed with EEG. This RCT will use fMRI, which can measure deep subcortical (and cortical) brain regions with high spatial resolution, to address for the first time with the use of a spatially well resolved imaging technique the question of the underlying mechanism of action of eTNS on the brain function of youth with ADHD. Therefore, the trial will be able to test not only if but also *how* and *why* eTNS works (if it does) on the brain of youth with ADHD. Last, this study has the power to explore treatment predictors including clinical, cognitive, and physiological measures such as easy-to-measure indicators of LC activity/arousal like heart rate or pupil diameter. Some of these easy to obtain measures may be able to identify a subgroup of youth with ADHD who respond optimally to the treatment which would be highly clinically useful for precision medicine.

The study will confirm whether eTNS is an efficacious non-drug treatment for ADHD symptoms that has minimal side effects and can be administered in-house and hence is likely to be preferred by youths, parents, and clinicians. Such a treatment would improve the healthcare and disease burden for youths with ADHD.

## Objectives

The main objective of this study is to evaluate whether eTNS over a 4 week period is efficacious in improving ADHD symptoms.

### Primary objective

To examine whether 4 weeks of nightly administration of real versus sham eTNS in youth with ADHD will improve weekly investigator-scored parent ratings of the ADHD-RS [14].

### Secondary objectives

To evaluate whether short term (four weeks) nocturnal real versus sham eTNS will:

2. Improve other measures of ADHD core symptoms measured at baseline, weekly during the four-week trial and at six months after randomisation:
  a. The child-rated Strength and Difficulties Questionnaire (SDQ) [15].
  b. The teacher-rated severity of ADHD symptoms as assessed by the Conners Teacher Rating Scale (Conners 3 T-S) [16] and the teacher rated ADHD-RS. [14]
  c. Emotional dysregulation, measured in the parent and child rated Irritability questionnaire, the Affective Reactivity Index (ARI) [17]
  d. The degree of mind-wandering as assessed by the self-rated Mind Excessive Wandering Questionnaire (MEWS) [18]
  e. Ratings of depression and anxiety using the revised child and adolescent depression scale rated by children and by parents (RCADS-25 and RCADS 25-P) [19]
  f. Ratings on the Columbia Suicide Severity Rating Scale (C-SSRS) [20]
  g. Cognitive performance in a range of executive functions including attention, inhibition, switching, and timing [21, 22]
3. Show sustained effects at six months in all primary and secondary outcome measures.
4. Have a good safety profile measured weekly during the trial and at follow-up in questionnaires of side effects adapted to eTNS rated by children and parents [9] and adverse events recording in the adverse events log.
5. Affect the sleep patterns of children with ADHD rated by parents using the parent-reported Sleep Disturbance Scale for Children (SDSC) [23].
6. Improve physiological measures,
  a) Heart rate; heart rate variability and objective hyperactivity measures tested in a wrist-hand device measured at baseline and post-treatment during the 3-4 h  of pre-assessment.
  b) Pupil diameter and head movement measured during rest and one of the cognitive tasks measured at baseline, post-treatment and at follow-up.
  c) Weight measured at baseline, post-treatment and at follow-up.
7. Mechanistic objective:

To assess the mechanisms of action of eTNS on brain activation in a subgroup of youth with ADHD between 10- and 18-years using fMRI on a 3 T scanner at the Institute of Psychiatry, Psychology & Neuroscience, London, during three fMRI tasks of working memory, motor inhibition and sustained attention [24–26] and a resting state measured at pre and post treatment timepoints.

## Methods

### Trial design

UK, two-centre, phase IIb, double-blind (participant, parent, postdoctoral Research Associate (RA) and analyst), parallel group, sham-controlled, superiority randomised controlled trial.

The study will be conducted across two centres to increase representativeness and higher rates of participant enrolment. The treatment duration will be four weeks as this has shown to be the optimal time period to elicit changes in ADHD symptoms in the pilot and pivotal studies [9, 12]. This is based on findings from the open 8-week trial where clinical changes were apparent in the first four weeks. Thus, 4 weeks was chosen to be a sufficient treatment length while balancing the aim to avoid drop-outs in this sham-controlled trial. A follow-up period of six months was chosen as this is standard for longer-term effects in studies of neurotherapies (and drugs) in ADHD [27–29].

### Participants, interventions and outcomes

#### Study setting

The study will be conducted at two academic institutions, the Department of Child & Adolescent Psychiatry/Social Genetic and Developmental Psychiatric Centre (SGDP), Institute of Psychiatry, Psychology and Neuroscience (IoPPN), at King’s College London (KCL), UK, and the Centre for Innovation in Mental Health (CIMH), School of Psychology, University of Southampton (SOTON), UK.

All fMRI scans will be completed at the 3T GE scanner at the Centre for Neuroimaging Sciences (CNS) at the IoPPN, KCL.

#### Eligibility criteria

Inclusion criteria

1. Children and adolescents, aged 8–18 years at study entry.
2. ADHD diagnosis (DSM-5 (TR); based on the Kiddie Schedule for Affective Disorders and Schizophrenia, for School-age Children- present and lifetime version, ADHD module (K-SADS).
3. A score higher than 24 on the investigator-scored parent-rated ADHD-RS (DSM-5) (to include participants who have relatively high symptoms).
4. Scoring above clinical cut-off for ADHD (5 or above) on the combined summary score of the child and parent ratings K-SADS [30].
5. Both parent and child need to speak sufficient English to complete study assessments.
6. IQ above 70 as assessed on the Wechsler Abbreviated Scale of Intelligence (WASI-II) [31] (to exclude participants with intellectual disability).
7. Participants should be either medication-naïve, or willing to come off their stimulant medication for one week before the trial or willing to be on stable stimulant medication for the duration of the trial.

Exclusion criteria

1. Comorbidity with any other major psychiatric disorder (except conduct/oppositional defiant disorder, mild anxiety and depression- as assessed on the K-SADS, as these are commonly associated with ADHD).
2. Alcohol and/ or substance abuse (as assessed on the K-SADS) (potential confound).
3. Neurological abnormalities, such as epilepsy (potential confound).
4. Current medication with atomoxetine or guanfacine in the past two weeks (as these have an effect on the arousal system thought to be improved with eTNS).
5. Participants who usully take drug holidays on weekends or holidays will not be able to participate in the study unless they are willing to take their stimulant medication in a stable way throughout the study or not at all throughout the study and 1 week before the study (Participants will be either on medication or off medication to decrease heterogeneity).
6. Implanted cardiac or neurostimulation systems (contraindication to eTNS).
7. Implanted metallic or electronic device in their head (contraindication to eTNS).
8. Presence of body worn devices (e.g., insulin pumps a t-VNS) (contraindication to eTNS).
9. Currently receiving any non-medical treatment (e.g., psychotherapy, counselling, parent-training, cognitive rehabilitation, EEG neurofeedback) (potential confound).
10. Participants with dermatitis (could be sensitive to patches).
11. Traumatic Brain Injury (TBI) (potential confound).

Additional exclusion criteria for the 56 patients that will participate in the fMRI study

12) Under 10 years old.
13) Have any MRI contra-indications (e.g., metal implants, pacemakers, braces, tattoos/piercings claustrophobia) which would render them unsuitable for the fMRI sub-study.
14) Be pregnant and/or breastfeeding if female.

#### Informed consent procedure

In order to minimise the need for face-to-face meetings, initial informed consent from parents, and from youth over 16 years old and assent from the child will be taken digitally by email or similar. Consent will be obtained by the postdoctoral RAs who will be trained in obtaining them. The postdoctoral RAs will share the information sheet on the screen and go through each section of the information sheet and also each section of the consent form. This allows for part of the eligibility assessments to be done at the participants’ convenience in their own home. The postdoctoral RAs goes through each section of the consent form and asks for an ink signature of the consent and assent forms on the first visit to the research centre.

#### Additional consent provisions for collection and use of participant data and biological specimens

Consent provisions are in place for the collection of participant data.

### Interventions

#### Explanation for the choice of comparators

It is common practice in brain stimulation studies to use sham stimulation as control condition [3, 32]. The placebo effect is particularly pronounced in studies that use technology and hence needs to be controlled for [32]. Subjects randomised to the sham group will receive a Monarch eTNS system identical in appearance and graphical user interface to the active stimulation device, but with the electrical stimulation routed through an internal resistor instead of the adhesive electrical patch after the first 30 s. This ensures that the rechargeable battery still drains appropriately and requires the subject to recharge it after each nightly therapy session, to maintain the study blind [9, 12]. Participants will be informed in a scripted presentation that “pulses may come so fast or so slowly that the nerves in the forehead might or might not detect a sensation”. They will also be told that most people do not feel the stimulation any more after some time (which is true). Like in the real eTNS condition, each night, parents (or youth) will turn on the device and press the up button until the stimulation is perceptible but not uncomfortable or painful or until the device reaches the maximum current (depending on the youth). Both the real and sham device will have a stimulation current between 0.2-10 mA.With sham, current will flow for 30 s every hour at a low frequency [33] and then be routed through the internal resistor. The scalp quickly adapts to stimulation and youths will not notice whether they receive the real or sham device [33].

eTNS is very simple to apply. The NeuroSigma company that develops the device will provide training to the trial manager at the start of the trial. In addition, the device manual will be provided to participants as well as a study specific guide with contact details for technical support.

#### Intervention description

eTNS will be performed with the Monarch eTNS System (NeuroSigma, Inc, Los Angeles, CA). The Monarch eTNS system received the European CE Mark in October 2015 for ADHD and FDA clearance in April 2019 as a medical device for the treatment of patients with ADHD. The European CE mark was not renewed in August 2021, and we hence applied and were granted approval from the Medicine and Healthcare products Regulatory Agency (MHRA) to conduct the study detailed in this protocol. The intervention will be applied every night for eight hours over four weeks.

The procedures for eTNS are based on well validated and safe methods used in the previous studies in ADHD [9, 12] as well as in other disorders such as adult depression, epilepsy, and post-traumatic stress disorder [34]. The stimulator is worn on the subjects’ pyjama/T-shirt and attached with thin wires to disposable, silver-gel, self-adhesive patch electrodes. Youth or parents of youth will apply a disposable patch across their/their child’s forehead to provide bilateral stimulation for ~ 8 h during sleep. The active condition will use 120 Hz repetition frequency with a 250 ms width, and a duty cycle of 30 s on and 30 s off. Stimulator settings will be established at baseline by titration in 0.2 mA increments ranging from 0 to 10 mA, which will identify a stimulation level which is perceptible but below the participants’ subjective level of pain/discomfort. Each night, parents or youth will turn on the device and press the up button to the maximum tolerable stimulation mA that is perceptible but not painful or uncomfortable or until the device reaches the maximum current (depending on the youth). The current that flows to the patch is limited to a safe range (maximum 10 mA). Once the optimal stimulation level is established for each participant, it will be turned on for 8 h during the night and will be 30 s on and 30 s off throughout.

#### Criteria for discontinuing or modifying allocated interventions

The parents and youth with ADHD will be in control of setting the nightly stimulation level and will be able to decide to vary this setting each night depending on how it is perceived that night. There will be weekly assessments to monitor any adverse events and side effects and technical support throughout if there are any issues or queries with the device. Clinical advice will be available if needed and if any concerns arise, a temporary discontinuation of using the device or a reduction of the nightly setting is initially recommended or a permanent discontinuation depending on the severity of the concerns.

#### Strategies to improve adherence to interventions

We will ask participants to record the length of time eTNS was applied each night in a nightly sleep diary as well as the nightly setting. We will emphasize compliance and the importance to be accurate when reporting usage of the device. Compliance will be defined in the Statistical Analysis Plan (SAP) (see Supplementary Material: Appendix 1).

#### Relevant concomitant care permitted or prohibited during the trial

Permitted medication: Participants will be allowed to be on stimulant medication for ADHD, provided they are on stable stimulant medication during the four weeks of the treatment, or they can come off their stimulant medication for two weeks before the trial and remain off during the four weeks treatment.

Prohibited medication: Participants will not be allowed to be on non-stimulant medication for ADHD such as atomoxetine and guanfacine since these medications may have similar effects to eTNS.

Non permitted care: Participants will not be permitted to have any other non-drug treatment such as psychotherapy, counselling, parent-training, cognitive rehabilitation, or EEG- neurofeedback during the treatment phase.

All concomitant medications and care are recorded in the database.

#### Provisions for post-trial care

No significant harm is anticipated for participants. However, should any serious adverse effects occur, then this would need post-trial care by the clinician on each site (SC, PS) who will see the youth/family and give advice.

## Outcomes

### Primary outcome measure

The primary outcome measure will be the Investigator-scored parent-rated ADHD symptoms measured on the well validated ADHD-RS at baseline and weekly during the 4-week trial [14]. The ADHD-RS recorded at the 6 months follow-up visit will be a secondary endpoint. The investigator scored parent-rated ADHD-RS is the most commonly used primary outcome measure in treatment trials of ADHD [6]. The postdoctoral RAs will be trained in obtaining this measure by the clinicians.

### Secondary outcome measures:

Secondary measures will be obtained at baseline, after four weeks of treatment and at six months follow-up except for side effects and sleep questionnaires which will also be assessed weekly during the 4-week treatment.

1. The teacher-rated ADHD symptoms assessed in the ADHD-RS (10 min) [14]
2. Teacher rated severity of ADHD symptoms using the Conners Teacher Rating Scale short form T-S (10 min) [16]
3. Self-reported outcome measure Strength and Difficulties Questionnaire (SDQ) (10 min) [15]
4. A scale that measures emotional dysregulation, the parent (ARI-P) and child (ARI-S) rated Irritability questionnaire (2 min) [17]
5. A questionnaire that measures the degree of mind-wandering rated by children (Mind Excessively Wandering Scale; MEWS) (5 min) [18]
6. Measures of depression and anxiety assessed in the child and parent rated short forms of the revised Child and Adolescent Depression scale (RCADS-25 and RCADS-25-P) (15 min) [19]
7. Measures of the Columba Suicide Severity Rating Scale (2 min) (C-SSRS) [20]
8. Parent-reported sleep quality measured in the parent-reported Sleep Disturbance Scale for Children (SDSC) (10 min) [23]
9. Executive functions: performance on a cognitive task battery developed for ADHD [21] including several executive functions using the following measures: (One hour)
  - Omission and commissions errors in the CPT sustained attention
  - Probability of inhibition in the GNG task
  - Simon RT effect for the Simon interference inhibition task
  - Errors in the time estimation task
  - Composite measures of mean reaction time, intrasubject standard deviation of reaction time and premature errors across the GNG, CPT and Simon tasks
  - Omission and commission errors in the Mackworth Clock vigilance task [22]
10. Physiological measures: This measure will only be measured before and after treatment. The specific measures are:
  - Heart rate, heart rate variability and objective hyperactivity measures will be measured in a wrist-held electronic device during pre and 4 week post-treatment visits conducted at the research center (3-4 h) (Electrodermal Activity, Heart Rate Variability, Movement Variability).
  - Pupil diameter (and head movement (coordinates of eye position) will be measured at rest for 1 min and during one of the computer tasks.
11. Safety measures: Safety will be assessed through weekly parent and children completed side effects rating scales adapted for eTNS [9] (3 min). In addition, there will be open questions, asking about general adverse events and their severity during the study period which will be recorded on the adverse events log and reported if needed.
12. Mechanistic objective: To assess the mechanisms of action of eTNS on brain activation in a subgroup of youth ADHD between 10- and 18-years using fMRI during three fMRI tasks of working memory (N-back task), motor inhibition (tracking stop task) and sustained attention [24–26] and a resting state scan (about 1 h) measured at pre- and post-treatment timepoints at a 3 T MRI scanner at the Centre for Neuroimaging Sciences (CNS) at the IoPPN, KCL.

Other measures which are not outcome measures include blood pressure at all visits, and an acceptability questionnaire to be filled in by parents at the end of the treatment and a blinding questionnaire for parents and youth as well as postdoctoral RAs asking them which device they thought was given to each participant after 1 week and after 4 weeks.

### Schedule of events

All participants follow the time schedule of assessments outlined in Table 1.

**Table 1:** Schedule of enrolment, interventions, and assessments

Enrolment/Assessments Timepoint	Eligibility at home/research centre	Baseline measure at home/research centre	Allocation	eTNS 1st week	eTNS 2nd week	eTNS 3rd week	eTNS 4th week/Endpoint at research centre*	6-month after randomisation Follow-up at research centre +
Consent form child (2 min)	x	x						
Consent form parent (2 min)	x	x						
Eligibility checklist (10 min)	x							
Background information (10 min)	x							
Kiddie Schedule for Affective Disorders and Schizophrenia (K-SADS) (1–1.30 h) (P)	x							
Kiddie Schedule for Affective Disorders and Schizophrenia (K-SADS) (50 min) (C)	x							
ADHD Rating Scale (ADHD-RS) (P) (10 min)	x	x		x	x	x	x	x
WASI-II (C) (40 min)	x							
ADHD Rating Scale (ADHD-RS) (T) (10 min)		x					x	x
Conners’ Teacher Rating Scale (Conners 3 T–S) (T) (10 min)		x					x	x
Strength and Difficulties Questionnaire (SDQ) (C) (10 min)		X					X	X
Edinburgh Handedness Inventory-short form (C) (1 min)	x							
Allocation			x				x	x
Columbia Suicide Severity Rating Scale (C-SSRS) (C) (2 min)		x					x	x
Affective Reactivity Index self-rating (ARI-S) (C) (2 min)		x					x	x
Affective Reactivity Index self-rating (ARI-P) (P) (2 min)		x					x	x
Mind Excessively Wandering Scale (MEWS) (C) (5 min)		x					x	x
Revised Children’s Anxiety and Depression Scale (RCADS-25) (P) (15 min)		x					x	x
Revised Children’s Anxiety and Depression Scale (RCADS-25) (C) (15 min)		x					x	x
Sleep disturbance Scale for Children (SDSC) (P) (10 min)		x					x	x
Sleep diary (2 min)				x	x	x	x	
Blinding Questionnaire parents/child/postdoctoral RAs (2 min)				x			x	
Acceptability survey (P) (C) (2 min)							x	
**Device training**								
Parents trained in device use (20–30 min)			x					
Use of device				x	x	x	x	
Trial Manager to check device use with family during week 1				x				
**Safety measures**								
Side effects (3 min) (C, P)		x		x	x	x	x	x
Adverse events log (3 min) (C, P)				x	x	x	x	x
Concomitant medication log (3 min)		x		x	x	x	x	x
**Lab measures for monitoring**								
Height, weight and vital signs (10 min)		x					x	x
**Neurocognitive measures**								
Go-no go task (5 min)		x					x	x
Continuous performance task (8 min)		x					x	x
Interference inhibition (Simon Task) (5 min)		x					x	x
Time estimation task (5 min)		x					x	x
Vigilance task (5 min)		x					x	x
**Physiological measures**								
Objective measure of heart rate, its variability and hyperactivity measure (wrist-held electronic device) to be used for 3-4 h during the pre and post treatment research visits		x					x	
Pupil diameter and head motion during rest and tasks		x					x	x
**fMRI measures in subgroup of 56 participants**								
Mock scan (can only take place at the research centre) (30 min)		x						
fMRI safety form (5 min)		x					x	
fMRI request form (10 min)		x						
Stop task (6 min)		x					x	
Sustained Attention task (12 min)		x					x	
Working memory task (6 min)		x					x	
Resting state fMRI (8 min)		x					x	
**Forms**								
Receipt of payment (1 min)	x	x					x	x

The following procedure will be followed in this study:

1. The participant will express interest in the study either in response to an advertisement, parent support groups, social media, schools or through information provided by their clinicians at CAMHS in London, Southampton or Portsmouth, GP practices or through Trust Consent for Contact (C4C) mechanisms.
2. The participant and the parents will then be sent a copy of the information sheet describing the research study.It contains comprehensive information about the study and the type of screening questions that they will be required to answer. They will have to provide consent to the study at the beginning of the first eligibility appointment that will be online. Consent will be provided by email in the first instance and in paper at the first visit to the research centre and be obtained by the Postdoctoral RAs at the site.
3. If the participant or the parent contacts one of the postdoctoral RAs (by telephone, digitally, email, or in person) to receive further information, s/he will be given the opportunity to ask any questions about the study, and to take more time to think about it if they so wish. They will be shown the eTNS device and how it functions. If they express interest in participating in the study, we will take their contact details to arrange a convenient appointment time.
4. Eligibility: Prior to the first testing session, participants will be reviewed online (via Teams) for eligibility and/or at the research centre. Their parents will also be asked to fill in some questionnaires on their youth’s ADHD behaviours (the ADHD-RS), background information and Edinburgh Handedness Inventory- short form [35]. Youths will also undergo an IQ test. Parents and youth will both be interviewed on the K-SADS. These sessions will also provide the youth and their parent with an opportunity to ask questions about the study and to familiarise themselves with the study protocol and the facilities. In addition, the youth can have a mock scan as well as MRI safety and request information during this visit to see whether they would like to take part in the optional fMRI scan.
5. Baseline: If the children are eligible to take part in the study, they will then have to come to the IoPPN, King’s College London or to the CIMH, University of Southampton, depending on where they live and have been recruited from, to do the first baseline assessment.

This will involve the following:

Performance of a computer test battery that measures attention, inhibition, and timing skills.

The children and their parents will also be asked to complete some more questionnaires about their behaviours (Parents and children: ARI, RCADS 25; children only: MEWS; SDQ, C-SSRS; parents only: SDCS). If participants prefer, they will be offered to fill in these questionnaires before they come to the Institute to shorten their visit. In addition, vital signs, height, and weight will be assessed.

The children and their parents will also be asked to request from their teachers to provide rating of the ADHD-RS and the Conners T-S. Efforts will be made to obtain teacher ratings; however, participants will be allowed to still be part of the study even if their teachers are unable to complete these measures.

They will be measured on physiological measures such as height, weight, heart rate, heart rate variability and objective hyperactivity measures in a wrist-held electronic device during the time of the research visit (i.e., ~  3-4 h).

Pupil diameter (percentage change from baseline measure) and head motion will be measured at rest and during one of the computerised tests.

All participants will be randomly allocated to an experimental (eTNS) and a control group (sham eTNS). The randomisation will be stratified by site, age, sex, and medication status. The TM will train the families on how to use the device and will follow up after the first night to ensure no further training is required for using the device.

A subsample of 56 children and adolescents will do the fMRI scan.

6. At the beginning of the trial, the postdoctoral RAs will explain the procedure in detail for how to use the eTNS device. Participants and their parents will be given instructions by the trial manager and two independent researchers on how to attach and switch on the eTNS battery device every night before they go to sleep. Both groups will have four weeks of treatment with the eTNS device for eight hours during each night. They will also be asked to fill in a sleep diary every morning over the four weeks which should record how many hours they had the eTNS attached as well as the nightly setting.
7. Week 1–3: Every week, the parents and children will have to complete the questionnaires on side effects, the parents will have to fill in the ADHD-RS. These weekly assessments will be conducted remotely. After the first week, the parents, children and postdoctoral RAs will also have to fill in a blinding questionnaire. Any adverse events and concomitant medication will be reviewed.
8. Endpoint at four weeks: At the end of the last week, the 4th, the parents will have to fill in several questionnaires about their children’s ADHD behaviours (ADHD-RS) and the other parent rated clinical questionnaires, the children will also be asked to fill in some questionnaires about their behaviour, and both will have to fill in the side events questionnaires. (Parents and children: ARI, RCADS 25; children only: MEWS, SDQ; C-SSRS; sleep diary. Parents only: SDSC. The children and their parents will also be asked to ask their teachers to provide again ratings of the ADHD-RS and the Conners 3 T-S. The children and their parents will have the option to fill in these questionnaires beforehand or remotely (at a maximum delay of three days before) to reduce the time spent at the research Institutes.

The children will also complete again the computerised tests of attention, inhibition, and timing to test whether they have improved in these skills after the training.

The blinding questionnaire will be repeated, and an acceptability survey will be given to parents and youth after the four week treatment.

They will be measured again in heart rate, heart rate variability and objective hyperactivity measures in a wrist-held electronic device during the pre- and post-treatment (4 weeks) research visits. Weight, and vital signs are also measured. Pupil diameter and head motion will be measured at rest and during one of the computerised tests. Any adverse event and concomitant medication will be reviewed.

The same subgroup as at baseline will do another fMRI scan.

9. Follow up at six months after randomisation: the parents will be asked to fill in (or answer by telephone, digital) the ADHD severity questionnaire (ADHD-RS), other clinical questionnaires and the child will have to fill in questionnaires about his/her behaviour (Parents and children: ARI,, RCADS; children only: MEWS, SDQ; C-SSRS; parents only: SDSC) and repeat the cognitive test battery one more time. Pupil diameter and head motion will be measured at rest and during one of the computerised tests. The teacher ratings (ADHD-RS and Conners 3 T-S) will be requested. Any adverse event and concomitant medication will be reviewed. In addition, side effects, vital sign and weight will be assessed.+
10. The participants will receive as reimbursement for their time £50 for the eligibility assessment, and for each of the assessments pre, post and at follow-up (i.e., £150). They will also receive £150 for the four weeks of treatment, i.e., in total £350.

For those who are taking part in the optional fMRI scan there will be an additional incentive of £50 for each scan (total £100). We will also reimburse their travel costs.

**Fig. 1:**
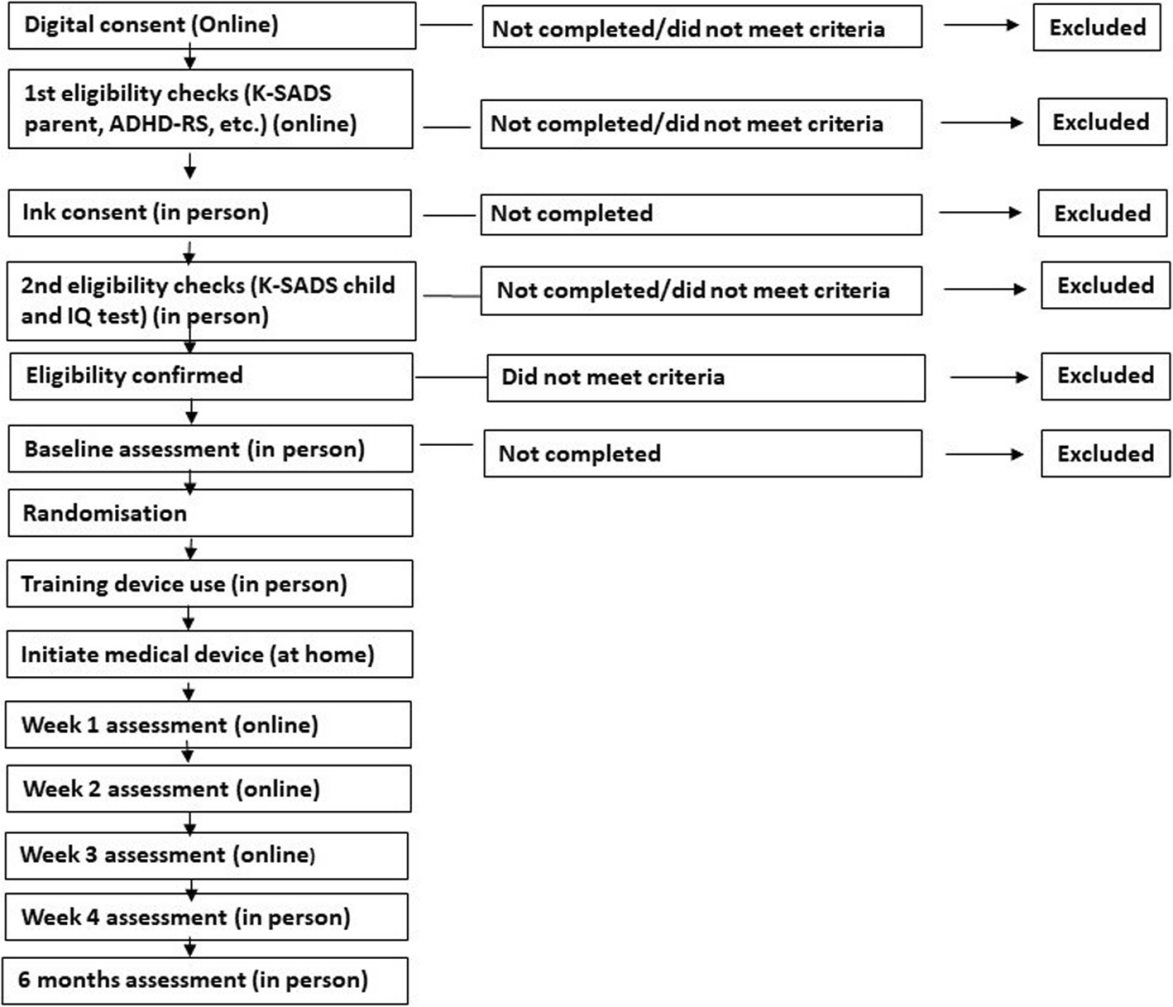
Schematic diagram of flow of participants (potential and actual) through the pre-trial assessment until the end of trial

See Fig. 1 for a Schematic diagram of flow of participants (potential and actual) through the pre-trial assessment until the end of trial.

### Sample size

The sample size is 150 youth with ADHD.

Based on the previous proof of concept trial [9], we anticipate a between group reduction in ADHD symptoms at four weeks between real eTNS versus sham eTNS with an effect size of 0.5 [9]. Using a baseline to post-treatment correlation of 0.5, with 90% power, and a 5% type I error, we estimate that we will need to recruit 128 participants (64:64). In order to account for a loss to follow up rate of 15% (which is a conservative estimate given our attrition rate of 9% for our fMRI Neurofeedback trial [29] and of 0% for our tDCS trial [28]), we have inflated the number needed to be randomised to 150 (75:75).

#### Using fMRI for the mechanistic outcome measure

We aim to detect a large between group effect of 0.7 (lower than the previously detected effect sizes of 2 and 2.4 based on a within-subject design on stimulant medication effects in the same Stop and sustained attention tasks, respectively; [36, 37] in a pre-post design, assuming a correlation of 0.7 (pre-post). In order to detect this difference with 90% power, at the 5% significance level we will need 44 participants. Assuming a loss to follow up of fMRI of 20% we will inflate the numbers included to 56.

## Recruitment

Participants will be included, recruited, and followed up at the IoPPN, King’s College London, and at the CIMH, University of Southampton in person and online assessments.

Participants will be recruited from London and surrounding National Health Service (NHS) and private clinics, from Southampton and Portsmouth clinics, parent support groups in London, Portsmouth and Southampton, GPs, advertisement at schools and social media. Some parent support groups are nationwide, and some families may travel a large distance to participate in the study. We will also utilise the SLaM Consent for Contact initiative to recruit Trust patients and will follow the related Trust policy. We will overrecruit participants who are stimulant medication-naïve or not currently taking stimulant ADHD medication or who are willing to come off their stimulant medication for one week before the trial and during the trial. For the optional fMRI part of the project, we aim to include as many participants as possible who are either stimulant medication-naïve or who do not currently take any stimulant medication or are willing to come off for one week before week before the study starts. However, we acknowledge that this may not be feasible, and some may be on stimulant medication.

### Assignment of interventions: allocation

#### Sequence generation, concealment mechanism, implementation

King’s Clinical Trials Unit (KCTU) will generate the allocation by minimisation by sex (male/female), medication status (on medication; off medication/naïve), site (London, Southampton) and age (8–13 years, 5 months; 13, 6 months to 19 years) using a bespoke validated online web-based system. A small proportion will randomly be allocated by simple randomisation to ensure allocation concealment. No data will be entered into the randomisation system unless a participant has signed a consent form to participate in the trial. Members of the research team will randomise a participant once all baseline measures are collected and eligibility confirmed. There will be a blind notification and confirmation that the participant has been randomised. An unblinded email will be sent to TM to allocate the device. All devices will be labelled with an individual Device Identification Number (DIN).

### Assignment of interventions: blinding

#### Who will be blinded

Participants, parents, postdoctoral RAs who assess the outcome, and the senior and junior trial statisticians as well as PIs will be blinded. The only non-blinded person is the trial manager, who will be unblinded throughout the trial. Two independent researchers who train the parents and participants in addition to the trial manager are also unblinded. The devices will be labelled with random numbers and the trial manager will keep a code as to which device number corresponds to group A or B. The devices look identical, and the instructions are identical for both sham and real eTNS. The TM will train parents and youth on how to use the device. The postdoctoral RAs at each location acting as outcome assessors will be fully blinded to the allocation. The Junior Trial Statistician will be blinded to the level of treatment until the first draft of the Statistical Analysis Plan (SAP) (see Supplement) is approved by the Trial Steering committee. After this point if required by the DMEC, they may be partially unblinded (data by unlabelled trial arms, denoted only A or B) or fully unblinded. The Senior Statistician will be fully blinded until after completion of the primary analysis. Following unblinding or partial unblinding of the junior statistician, any required changes to the SAP will be made by the senior statistician only.

In the previous study of eTNS in ADHD, some but not all subjects in the active and sham groups reported feeling some sensation, which generally faded with time. In the previous trial, blinding was successful. There was no difference between groups on questions pertaining to belief in having active or sham device [9]. The blinding was also successful in similar studies with the device in depression [38]. To further enhance the blinding in this study, the first 30s of every hour of stimulation will be real stimulation but at a lower frequency in the sham eTNS and then be switched off. The scalp adjusts very quickly to the stimulation. and the switch-off is not noticeable.

To further protect the blinding, subjects are counselled during enrolment that stimulation may not be perceptible, and that most people do not feel the stimulation after some time because the scalp adapts to the stimulation.

To assess the success of the blinding, youths, parents and postdoctoral RAs will be asked to guess which study device participants have been using through a blinding questionnaire at each participants’ week one visit and at the end of treatment at week four.

A series of precautions will be implemented to ensure the integrity of the blinding process:

1. the sham devices are programmed to have a 30s stimulation at the beginning of every hour at a lower frequency than the real stimulation. This is to avoid making it obvious to the participants which device they have been allocated.
2. the postdoctoral RAs will input the relevant participant’s information into the KCTU randomisation system that randomises the participant to either real or sham treatment.
3. the families will be trained using the device by the TM. Families are told that the sensory experience may vary from person to person, that some participants do not feel anything, and that prior users have used words like “tingling,” “vibration,” “buzzing,” and “tickling” to describe their experiences. They will also be told that most people do not feel anything after some time because the scalp adapts.
4. participants are instructed to not discuss the experience of using the device with any member of the research team.
5. a device manual will be given to the families on how to use the device and a study specific guide. If any queries arise there will be a separate email address and phone number for technical support.
6. technical support given to participants will be given at first instant by the trial manager who is unblinded but will follow a pre-set guide regarding support and advice to families.
7. members of the research team will remind participants to not discuss any issues regarding the devices apart from how many hours they used it each night.
8. the nightly setting (intensity) of the device is kept from the research team and will only be entered into the database prior to database lock.
9. all assessments will be conducted by blinded staff (postdoctoral RAs at each site will be blinded).

### Procedure for unblinding if needed

Participants will only be unblinded if there are Serious Adverse Events (SAE). This can be done by the TM and the other two researchers that train participants in the device, all of whom are unblinded.

### Data collection and management

#### Plans for assessment and collection of outcomes

The postdoctoral RAs will be trained in obtaining the investigator-scored parent-ratings of the ADHD-RS (primary outcome) by Professor Cortese and Professor Santosh. Professor Rubia will provide training for administering the neurocognitive tasks and the fMRI tasks. User manuals will be developed to ensure consistent procedures for device training, cognitive assessments and obtaining physiological measures. See SPIRIT Table 1 for a summary of the schedule of enrolment, intervention and assessments.

#### Plans to promote participant retention and complete follow-up

Efforts will be made to retain all the participants recruited into the trial. It will be emphasized to the participants that they should only agree to take part in the trial if they think that they would be able to commit to participating until the follow-up visit at six months. Postdoctoral RAs will be as flexible as possible and assist participants with the travel arrangements to the visits to the research centres, if needed. Visit assessments and weekly assessments over the phone and online will be booked for a day and time that suits the participants. It will be stressed to the participants that it is important not to have missing values and if no research visits can be arranged, obtain, if possible, at the minimum the primary outcome rating scale. Participants will be reimbursed after each research visit.

There is little evidence of side or (serious) adverse effects of eTNS [38]. Hence, withdrawal from the treatment due to adverse effects is not expected. Nevertheless, potential adverse effects have been listed in the participants information sheet, which will be provided to participants and their parents for their information. Side and adverse effects will be assessed every week of treatment assessment. Participation in the treatment will be discontinued if the participant decides they no longer wish to continue, or if it is recommended by the research team. Participants will have the right to withdraw from the treatment or from data collection at any time for any reason. Withdrawal from treatment will not constitute withdrawal from data collection unless the participant also wishes to withdraw from data collection. Participants who wish to withdraw from the treatment will be asked to confirm whether they are still willing to continue to provide data. All efforts will be made to continue to obtain follow up data if the participant is willing. Reasons for withdrawal from treatment or from data collection will be reported.

### Data management

The custodian of the trial data will be Professor Katya Rubia and the data management will follow King’s College London guidelines. The trial will adhere to the Data Protection Act 2018 and GDPR regarding the collection, storage, and processing of the study data. The trial data will contain demographic data, KSADs voice recording data, clinical assessment and questionnaire data, behavioural outcome data, cognitive performance data, physiological data, heart rate data, eye-tracking data, and fMRI data in electronic format and password-protected. The data from the E4 Empatica wristband will be pseudo-anonymised (dummy information such as dummy name and email addresses). For the Tobii Pro eye-tracker only a PIN will be entered onto these platforms.

The King’s Clinical Trial Unit (KCTU) at King’s College London will provide an InferMed Macro 4 system as a web-based database as well as a randomisation system. The data entered will be in linked-anonymised format with a full audit trail which automatically stamps all data entry and changes. These systems will be maintained by KCTU for the project's length and are hosted by KCL servers. Any changes to the database will be agreed to by the Trial Management Group (TMG) and the data will be updated by the postdoctoral RAs. No changes can be made to the randomisation system, but notes can be added to the participant in case of any errors.

Access to the database and randomisation system will be restricted by user specific passwords and these can only be requested by the CI or nominated person. Only participants’ month and year of birth and initials will be entered into the database and only after participants have signed a consent form.

The trial manager and junior statistician will undertake appropriate reviews of the entered data and any queries will be resolved by raising Data Clarification Request (DCR). The primary outcome, the investigator-scored parent-ratings of the ADHD-RS, will be 100% Source Data Verification (SDV) checked, and all other data will be SDV checked at a minimum of 80% by the TM.

At the end of the trial, data entry access will be removed for the Postdoctoral RAs once all data queries have been resolved. A final data extract will then be requested and checked to ensure there are no further changes before database-lock. A blinded dataset will at this stage be provided by the KCTU and only once the primary analysis has been completed, will unblinded information be requested. A statistical analysis report will be prepared by the statisticians for the TMG.

FMRI data will be acquired according to standard protocols, and subject to on-going calibration and quality control procedures within the CNS at the IoPPN. fMRI imaging data will be linked by ID to clinical, demographic, and neuropsychological data. For all the processing currently envisaged, data will be processed and stored in NifTI format (http://nifti.nimh.nih.gov/), allowing it to be easily read by most current neuroimaging research packages, allowing data-sharing, and ensuring long-term data validity.

## Confidentiality

When consent forms are signed, a copy will be provided to the participant, and the original will be retained in the Investigator Site File separate from any other research data. Participant initials and date of birth (month and year only) will be entered into the Macro database and randomisation system. Personal information will only be collected and accessible by the research team for the duration of the trial. This data will be stored separately from the research data.

### Statistical methods

#### Statistical methods for primary and secondary outcomes

A full statistical analysis plan will be drafted in accordance with the KCTU Standard Operating Procedures authored and reviewed by the Trial Statistician (DS) and the Senior Statistician (BC), respectively, and approved by the Trial Steering Committee. The SAP presents a clear and structured plan for the primary outcome, required data manipulation, and analysis (see Supplement). All changes to the SAP after approval by the TSC will be authorised by a statistician who is fully blind (BC).

For the fMRI data a statistical analysis plan will be published on open science platforms.

#### Primary outcome analysis

The primary analysis will be conducted using a mixed-effects linear model, fitting 4-week ADHD symptoms scores, each participant will be fitted as a random intercept, and adjusted for fixed effects of baseline ADHD score, site, age, sex, and medication. We will estimate 95% confidence intervals, two-sided p-values and effect sizes for the between group post-baseline difference. Alpha will be set at 0.05.

#### Secondary analyses

Secondary outcomes of continuous data will be analysed consistently with the primary outcome. Dichotomous secondary outcomes will be analysed using a logistic regression, adjusted for the same covariates as within the primary outcome.

#### Longer-term follow up

All outcomes will be repeated at 6 months follow up. These will be analysed with linear and logistic regression analyses. The outcome will be the 6-month time-point, and these will be adjusted by: baseline score, site, age, sex, and medication.

#### Populations under investigation:

A modified intention to treat (ITT) population will include all participants randomised with at least one post baseline weekly assessment of the primary outcome. The primary analysis will use the ITT population.

#### Interim analyses

There is no planned formal interim analysis to assess efficacy within the study. However, trial progress will be presented to the Trial Steering Committee (TSC) and Data Monitoring and Ethics Committee (DMEC) following the TSC terms of reference and DMEC Charter.

At 19 months after start of the project (15 month after recruitment started) after the first site is opened to recruitment, an internal pilot will be carried out to demonstrate feasibility of the study. The following progression criteria will be presented to the (DMEC).

**Table:** 

Recruitment	Red < 60%	Amber (60–80%)	Green > 80%
Recruitment of (*n* = 75)	< 45	45–60	> 60

The DMEC will review the target sample size based on recruitment, missing data, and variance of outcome variables.

### Methods for additional analyses (e.g., subgroup analyses)

#### Sensitivity analysis

A sensitivity analysis of the primary outcome will be carried out to estimate the treatment effect in those participants who complied to the intervention, using a per-protocol/complier average causal effect analysis.

#### Mechanistic analysis

Structured mediation analyses will be conducted to explore potentially mediating effects of brain function and cognitive measures on the clinical outcome.

Logistic regression models will be used to test predictors of response based on clinical, imaging, cognitive and physiological (heart rate and pupil diameter) measures using a reduction of 20% or more in clinical ADHD-RS symptoms as a typical indicator of treatment response as per NICE guidelines (https://www.nice.org.uk/guidance/ng87).

For fMRI analyses in a subgroup of 56 participants, non-parametric FSL RANDOMISE analysis package will be used (http://www.fmrib.ox.ac.uk/fsl/). Functional connectivity analyses will use thalamus, basal ganglia, and lateral and medial frontal regions as seed regions to test our hypothesis that task-relevant fronto-striato-thalamic networks will be up-regulated with eTNS. We will also use LC as seed region for our hypothesis that LC-frontal connections will be increased with eTNS. For the resting state fMRI analysis, we will use regions of interest of the default mode network (reflecting mind-wandering), and of dorsal and ventral attention and cognitive control networks to test our hypothesis of increased functional connectivity in these networks with eTNS and of the increased anti-correlation between these networks and the default mode network.

#### Methods in analysis to handle protocol non-adherence and any statistical methods to handle missing data

Missing baseline data will be negligible. If we do observe missing data, we are following an approach set out to handle missing data by Jakobsen [39]. We will use longitudinal models for the primary analysis using maximum likelihood estimation, and baseline predictors of missingness will be included as covariates. If post-baseline variables are predictive of missingness, inverse probability weighting will be used. Thus, the primary analysis will be unbiased under a missing at random assumption.

As recommended by Jacobsen [39], in the presence of missing data, we will carry out sensitivity analyses looking at the best- and worst-case scenarios for the theoretical range of unobserved missing values under a missing not at random assumption. This approach is also consistent with ICH E9 (https://www.ema.europa.eu/en/documents/scientific-guideline/ich-e9-r1-addendum-estimands-sensitivity-analysis-clinical-trials-guideline-statistical-principles_en.pdf). This is outlined in the Statistical Analysis Plan (see Supplement).

#### Plans to give access to the full protocol, participant level-data and statistical code

A data sharing dataset will be created from the raw data by the study analyst, which will not include participant initials, date of birth or any other identifiable data and study PIN will be altered so that individuals are not recognisable from the dataset. Access to the dataset can be granted after approval by the CI and PI once all planned publications have been completed.

The study will comply with the General Data Protection Regulations (GDPR).

### Oversight and monitoring

#### Composition of the coordinating centre and trial steering committee

The project will be led by Chief Investigator (CI) Professor Rubia and supported by Trial Manager (TM), Lena Johansson both based at King’s College London. Weekly meetings will be held online to discuss recruitment, assessments, and any other issues. These meetings are attended by CI, Principal Investigator (PI) for the Southampton site, Professor Samuele Cortese (University of Southampton), TM and postdoctoral RAs.

The Trial Management Group (TMG) which consist of all co-applicants, TM, Junior Statistician and postdoctoral RAs will meet monthly to monitor all aspects of the trial progress.

The Data Management team will consist of the CI, PI, Professor Ben Carter (Senior Statistician), Dominic Stringer (Junior Statistician) and TM.

The Trial Steering Committee (TSC) will meet every 6–12 months remotely. TSC will report to the NIHR EME Programme if requested. Its purpose is to provide overall supervision of the trial, approving the protocol and amendments, monitoring adherence to the protocol, and providing independent advice on all aspects of the trial. Observers from the NIHR EME Programme will be invited to all TSC meetings.

#### Composition:

1. It will be chaired by an independent senior clinician chair.
2. Two further independent clinical academics.
3. An independent trial statistician.
4. PPI: a mother with ADHD with a child with ADHD.
5. The lead investigators of each site will attend as part of the non-independent members (Profs Rubia and Cortese).

#### Composition of the data monitoring committee, its role and reporting structure

DMEC will meet every 6–12 months (depending on the stage of the study) before the TSC meeting using remote meetings. It will monitor the safety, ethical conduct, protocol deviations and quality of the data. It will have access to all trial data and will receive regular reports on adverse events. The role of the DMEC is to review the accruing trial data and to assess whether there are any safety issues that should be brought to participants’ attention or any reasons for the trial not to continue. The DMEC will be independent of both the investigators and the funder/sponsor and will be the only body that has access to unblinded data if needed. It will make recommendations to the TSC. The independent DMEC can advise discontinuation of the trial, e.g., because of safety concerns about the trial. DMEC will have access to the database detailing all Adverse Events (AEs) and Serious Adverse Events (SAEs). Membership of the DMEC will be independent of the applicants and of the TSC. The DMEC will be notified of any serious adverse events (which are unlikely) as they occur and will consider whether any interim analyses are warranted, review data and advise the TSC on any ethical or safety reasons why the trial should be prematurely ended. Administrative support to the DMEC will be provided by the TM and junior statistician (DS).

#### Composition:

1. It will be chaired by an independent international expert experienced in conducting clinical trials with child mental health populations.
2. It will also comprise an independent senior trial statistician.
3. Another independent senior child and adolescent clinician

#### Adverse event reporting and harms

AE and Adverse Device Effects (ADE) will be recorded weekly during the treatment in free report and side effects will be recorded weekly in side effects questionnaires. All adverse events/ side effects that need attention at any time will be flagged by the post-doctoral RAs and reviewed by the relevant local clinician/child psychiatrist (Prof Cortese in Southampton and Prof Santosh in London). In addition, all adverse events and side effects will be reviewed weekly by the team meeting and monthly by the Trial Management Group (TMG) meetings.

SAEs are very unlikely but will be reported to the main REC and to MHRA should they occur within 7 days and participants will then be unblinded.

All SAEs, Suspected Serious Device Effect (SADE) and Unanticipated Serious Adverse Device Effect (USADE) (excepting those specified in this protocol as not requiring reporting) will be reported immediately to the Chief Investigator and to the Sponsor, REC and MHRA.

#### Frequency and plans for auditing trial conduct

It is the responsibility of the sponsor (King’s College London and South London and Maudsley NHS Foundation Trust) to conduct audits, but these are not expected for this trial.

#### Plans for communicating important protocol amendments to relevant parties (e.g. trial participants, ethical committees)

Any protocol amendments will require approvals from the MHRA and REC/HRA. Any protocol amendments that trial participants will need to be made aware of will be communicated via updated information sheets.

#### Dissemination plans

The results of the study will be reported and disseminated at international conferences and in peer-reviewed scientific journals, ADHD Guideline groups, and to the public via media communications, social networks, presentations at science festivals, charities, parent and patient support groups, and clinics. Findings will also be disseminated via the KCL website https://www.kcl.ac.uk/research/attens-project and a dedicated google website https://sites.google.com/view/kcl-attens/home

## Discussion

The primary aim of this study is to test the efficacy of eTNS in improving ADHD symptoms in children and adolescents with ADHD in a confirmatory, multi-centre, parallel-arm, double-blinded (postdoctoral RAs, parents, and youth), sham-controlled randomised trial. We furthermore want to test whether eTNS treatment improves other related clinical measures, executive function performance, objective hyperactivity measures, whether it is safe; whether effects on all outcome measures persist at six months follow-up; whether we can establish predictors of treatment efficacy and to understand the underlying mechanism of action on ADHD brain function using fMRI.

Given the findings of the previous pilot trial in the USA in 62 ADHD children between 8 and 12 years, which showed an improvement of ADHD symptoms with a medium effect size after four weeks of nightly eTNS, we expect to replicate clinical efficacy on ADHD symptom reduction with medium effect size in a much larger, multicenter trial. Furthermore, we expect that we will find the same beneficial clinical effect in a larger age range between 8 and 18 years, including not just children as in the previous trial, but also adolescents. If we can show clinical efficacy of eTNS on ADHD symptom reduction in adolescents, then this would be highly relevant as there is very low compliance in adolescents with ADHD of taking pharmacological treatment such as stimulant medication [6, 7].

If efficacious, then a non-pharmacological treatment might be preferred by parents and youth [40]. There has been an exponential worldwide increase in stimulant prescription over the last decades. However, stimulants often have side effects on sleep, appetite, irritability, nausea/vomiting, abdominal pain, headaches, labile mood and growth suppression, even though they can be transient. They are also controversial due to abuse and diversion potential [6]. Also, only 50% of participants tolerate it sufficiently, and they are indicated with caution for certain comorbid conditions (such as cardiovascular malfunctions, sleep problems) and adherence is poor, in particular in adolescence [6, 7]. Last, there is evidence for drug tolerance, as shown in studies using positron emission tomography [41, 42], which could explain why efficacy is larger in short-term studies, with relatively smaller effect sizes in meta-analyses of studies over 12 weeks [6] and little evidence for longer-term efficacy based on epidemiological and follow-up studies [42].

Other non-pharmacological treatments such as behavioural therapies, cognitive training (CT) [38], Electrophysiology-Neurofeedback [3], non-invasive brain stimulation, fMRI Neurofeedback [27, 29] or dietary interventions show only small to moderate efficacy on improving ADHD symptoms or cognition [3–5]. eTNS is hence the non-pharmacological treatment that has shown the largest effect size so far in improving ADHD and is the only non-pharmacological medical device licensed as treatment for ADHD by the FDA.

We furthermore will also test for potential improvements in cognition. The open label trial of eight weeks of eTNS treatment showed improvements in executive functions [12] and the larger RCT [9] showed that there was an association between lower working memory and spelling and mathematical skills and response to eTNS [43]. We therefore expect in this study to show improvements in executive and other functions that are typically abnormal in ADHD [4, 21] by using a relatively large battery of the most ADHD-relevant tasks including executive and non-executive functions such as motor and interference inhibition, sustained attention, vigilance, and time estimation.

Compared to the previous trials, where the sham condition did not deliver any stimulation, we have improved the blinding in this trial by giving participants in the sham condition 30 s stimulation every hour of nightly device use. We believe this will substantially increase blinding. We furthermore will not disclose the intensity of stimulation to the blinded postdoctoral RAs.

Like most treatments, eTNS is likely to improve ADHD symptoms in a particular subgroup of youth with ADHD. It will be crucial to understand which clinical, cognitive, or neurophysiological parameters predict treatment response. For this purpose, we will test for treatment response prediction using our large baseline datasets of clinical, cognitive and fMRI measures. In particular, parallel to the previous findings of lower cognitive skills in working memory and lower activity over right frontal areas being predictive to response to eTNS [43], we hypothesise that lower right frontal activation in fMRI during the resting state and the three fMRI tasks as well as lower performance on the ADHD-relevant tasks including the fMRI working memory task will be predictive of eTNS response.

There is currently very little knowledge on how eTNS works in the brain in general and how it works in the brain of youth with ADHD. Trigeminal afferent fibers carry sensory information of the face and project to the nucleus solitarius, the locus coeruleus (LC), a small brainstem region consisting of neuroadrenergic neurons that is the main source of noradrenaline (NE) projection in the brain, the raphe nuclei (the sole source of serotonin projection to the rest of the brain), the reticular activation system (RAS) (crucial for arousal) and thalamic structures, and from there to cortical, limbic and other subcortical structures [10].

There is evidence for a close connection between the trigeminal system and in particular the RAS and LC [8]. The rate of NE release from the LC influences arousal and modulates other neurotransmitters such as dopamine and glutamate that mediate attention. The LC via the nucleus tractus solitarius also connects the autonomic nervous system (ANS) from which it receives signals, and which regulates bodily functions (i.e., heart rate, respiration, perspiration, and pupil dilation) with the rest of the brain which is the mechanism by which arousal impacts on attention. Pupil dilation and diameter in particular has been shown to be an indicator of LC activity and arousal [8, 44, 45], which is the reason we test pupil dilation in this study. Thus, the LC has widespread, reciprocal connections with prefrontal cortex (PFC) regions (in particular anterior cingulate cortex (ACC), orbitofrontal cortex (OFC) and ventromedial prefrontal cortex (vmPFC), parietal attention regions, insula, thalamus, hypothalamus and amygdala [8, 45]. Through its connections with the autonomic and central nervous systems, in particular via their specific connections to thalamo-cortical activation [46] and via NE projections the LC and RAS modulate arousal [8, 45] and thus play a role in vigilance, sustained attention, working memory, self-regulation and response to salient stimuli [46, 47], partly by modulating the availability of dopamine and glutamate at task-relevant sites. All of these functions have been found to be impaired in children and adults with ADHD [2, 4]. The LC-NE system modulates the PFC, enhancing signal-to-noise ratio via increased stimulus-related phasic and decreased tonic LC activity, which improves in particular sustained attention, vigilance and the ability to inhibit distraction and therefore optimises performance [8, 45]. It has therefore been argued that the LC-phasic response and concomitant NE release provides a temporal attentional filter that facilitates task-related behaviour, inhibits distraction, and therewith improves cognitive performance. The ANS, brainstem, and cortical systems are thus closely interconnected in their mediation of the regulation of behaviour and cognition [48].

Studies have shown improvements in pain, in neurological disorders such as epilepsy, migraine as well as in psychiatric disorders [38]. Our meta-analysis of TNS studies in neurological and psychiatric disorders showed improvement in migraine pain combined with medication and in depressive symptoms across three disorders [38].

Despite these interconnections between the trigeminal system, the arousal system and cortico-limbic brain structures and evidence for improvement of symptoms in some neurological and psychiatric disorders, relatively few imaging studies have tested effects of eTNS in humans. The previous trial in ADHD used quantitative EEG and found an increase in broadband power over right inferior and frontal midline regions with qEEG that correlated with improvements in clinical ADHD and hyperactive-impulsive sub-symptoms, suggesting mediation of clinical effects [9]. A study using somatosensory evoked potentials found increase in indices of thalamo-cortical activity [49]. However, EEG has relatively poor spatial resolution and can only measure cortical brain regions while eTNS has prominent effects on subcortical areas. Studies using PET found that eTNS increased metabolic activity in ACC, limbic regions, inferior, medial, and orbitofrontal cortices [50, 51]. fMRI studies found increased activity after eTNS in ACC, insula, thalamus, hypothalamus, cerebellum as well as brainstem in healthy, migraine or pain participants [51–53]. Therefore, the effects of eTNS on arousal via the LC/NE system and its connections to other thalamo-cortical, limbic, frontal, and parietal brain systems and via its modulation of dopamine and other neurotransmitters that are crucial for attention and self-regulation is a very plausible mechanism of action for improvement of attention and self-regulation symptoms in ADHD. The fMRI analysis included in the study will provide unprecedented knowledge on the mechanisms of action of eTNS on the brain activation in children with ADHD.

For practical reasons and in line with advice from public and patient user groups we consulted on the study, we will include medicated as well as unmedicated participants. This was both practical and ethical, to be able to recruit sufficient numbers of participants and for youth not to have to withhold their medication for four weeks. However, potential stimulant medication effects on eTNS are unknown and could present a confound. We hope to be able to conduct a sensitivity analysis of only unmedicated youth to clarify potential medication effects.

The study will be the first large multicentre trial to establish whether eTNS is an effective non-drug medical-device based treatment for ADHD with minimal side effects that can be administered in-house and is therefore likely to be preferred by the children and their parents. The study will also provide unprecedented evidence on the underlying mechanisms of action on the brain function in ADHD. Such a non-pharmacological treatment with minimal side effects would improve the healthcare and disease burden for patients with ADHD.

## Trial status

The trial is currently recruiting. Recruitment started in September 2022 and will last until the end of December 2024.

### Supplementary Information

**Supplementary Material 1.**

## Data Availability

The CI, PI, TM, postdoctoral RAs and the Junior and Senior Statistician will have access to the final dataset to analyse the data and for reporting the findings. There are no contractual agreements in place to restrict this access. Once the results of the trial have been reported, a data sharing dataset will be available.

## Abbreviations

ACC: Anterior Cingulate Cortex
ADHD: Attention-Deficit/Hyperactivity Disorder
ADHD-RS (P): ADHD Rating Scale - Parent
ADHD-RS (T): ADHD Rating Scale - Teacher
ADE: Adverse Device Effects
AE: Adverse Events
ANS: Autonomic Nervous System
ARI (P): Affective Reactivity Index - Parent
ARI (C): Affective Reactivity Index - Child
C4C: Trust Consent for Contact
CAMHS: Child and Adolescent Mental Health Service
CI: Chief Investigator
CIMH: Centre for Innovation in Mental Health
Conners 3 T-S: Short Forms of the Teacher Rating Scales
CNS: Centre for Neuroimaging Sciences
CPT: Continuous Performance Task
C-SSRS: Columbia- Suicide Severity Rating Scale
DCR: Data Clarification Request
DIN: Device Identification Number
DMEC: Data Monitoring and Ethics Committee
DSM-5 (TR): The Diagnostic and Statistical Manual of Mental Disorders, Fifth Edition, Text Revision
EDC: Electronic Data Capture
EEG: Electroencephalography
EME: Efficacy and Mechanism Evaluation
eTNS: External Trigeminal Nerve Stimulation
FDA: USA Food and Drug Administration
fMRI: Functional Magnetic Resonance Imaging
GDPR: General Data Protection Regulation
GNG: Go No Go Task
GP: General Practitioner
HRA: Health Research Authority
IoPPN: Institute of Psychiatry, Psychology & Neuroscience
ITT: Intention to Treat
KCL: King’s College London
KCTU: King’s Clinical Trials Unit
K-SADS: Schedule for Affective Disorders and Schizophrenia, ADHD module
LC: Locus Coeruleus
NE: Noradrenaline
NIHR: National Institute for Health Research
MEWS: The Mind Excessive Wandering Scale; a mind-wandering questionnaire rated by children
MHRA: Medicine and Healthcare Products Regulatory Agency
NHS: National Health Service
OFC: Orbitofrontal Cortex
PFC: Prefrontal Cortex
PPI: Patient and Public Involvement
QEEG: Quantitative Electoencephalography
RA: Research Associate
RAS: Reticular Activation System
RCADS-25 and RCADS-25-P: Revised Child and Adolescent Depression scale short form child and parent rated
RCT: Randomised Controlled Trial
REC: Research Ethics Committee
SSDE: Suspected Serious Device Effect
SAP: Statistical Analysis Plan
SDSC: Sleep Disturbance Scale for Children (parent rated)
SDQ: Strength and Difficulties Questionnaire
SDV: Source Data Verification
SGDP: Social Genetic and Developmental Psychiatric Centre
SLaM: South London and Maudsley NHS Foundation Trust
TBI: Traumatic Brain Injury
tDCS: Transcranial Direct Current Stimulation
TM: Trial Manager
TMG: Trial Management Group
TSC: Trial Steering Group
tVNS: Transcutaneous Vagal Nerve Stimulation
USADE: Unanticipated Serious Adverse Device Effect
vmPFC: Ventromedial Prefrontal Cortex
WASI-II: Wechsler Abbreviated Scale of Intelligence
