# Supplementary material for: The efficacy of real versus sham external Trigeminal Nerve Stimulation (eTNS) in youth with Attention-Deficit/Hyperactivity Disorder (ADHD) over 4 weeks: a protocol for a multi-centre, double-blind, randomized, parallel-group, phase IIb study (ATTENS)
Source: BMC Psychiatry. 2024 Apr 30;24:326. doi: 10.1186/s12888-024-05650-1 (PMC11059677; doi:10.1186/s12888-024-05650-1)
Supplement: Supplementary file 1 — Supplementary Material 1. [file 12888_2024_5650_MOESM1_ESM.docx]

**Supplementary Material: Appendix 1.**

**ATTENS Trial: A multi-centre, double-blind, randomized, parallel-group, phase IIb study to compare the efficacy of real versus sham external Trigeminal Nerve Stimulation (eTNS) on symptoms in youth with Attention-Deficit/Hyperactivity Disorder (ADHD)**

Statistical Analysis Plan

Version 1.0

17/10/2023

Authors:

Dominic Stringer, Prof Ben Carter

**1. PURPOSE AND SCOPE OF STATISTICAL ANALYSIS STRATEGY**

This document details the presentation and analysis strategy for the primary papers reporting results from the ATTENS trial. It is intended that the results reported in these papers will follow the strategy set out herein; subsequent papers of a more exploratory nature will not be bound by this strategy but will be expected to follow the broad principles laid down for the principal paper(s). These principles are not intended to curtail exploratory analysis or to prohibit sensible statistical and reporting practices, but they are intended to establish the strategy that will be followed as closely as possible, when analysing and reporting the trial. Reference was made to the trial protocol (v7.1

v.7.1 06.03.24), ICH guidelines on Statistical Principles (E9) (1)and CONSORT (2) guidelines.

This trial is being conducted by KCTU and this Statistical Analysis Plan (SAP) has been developed following KCTU Statistics Standard Operating Procedure ST-02.

**2. DESCRIPTION OF THE TRIAL**

**2.1 Key Trial Personnel**

**Chief investigator:**

Prof Katya Rubia

**King’s Clinical Trials Unit (KCTU) Trial statisticians:**

Trial Statistician: Dominic Stringer

Senior Statistician: Ben Carter

**Trial manager:**

Lena Johansson

Funding is from the NIHR EME Programme (NIHR130077).

**2.2 Principal research objectives to be addressed**

***The primary aim*** of the ATTENS Trial is to examine whether 4 weeks of nightly administration of real versus sham eTNS in ADHD children will improve weekly investigator-assessed parent ratings of the ADHD-RS.

***Secondary objectives*** are as follows***:***

To evaluate whether short term (4 weeks) stimulation of nocturnal real eTNS versus sham eTNS will:

1. Improve other measures of ADHD core symptoms and related measures, measured at baseline, after 4 weeks and at 6 months after randomisation:

a. the child-rated Strength and Difficulties Questionnaire (SDQ)

b. Teacher-rated severity of ADHD symptoms as assessed by the Conners Teacher Rating Scale (Conners 3 T-S) (Conners 2008, Conners et al.,2011) and the teacher rated ADHD-RS

c. Emotional dysregulation, measured in the parent and child rated Irritability questionnaire (Affective reactivity Index; ARI)

d. The degree of mind-wandering as assessed by the self-rated Mind Excessive Wandering Questionnaire (MEWS)

e. Ratings of depression and anxiety using the revised child and adolescent depression scale rated by children and by parents (RCADS-25 and RCADS 25-P)

f. Ratings on the Columbia Suicide Severity Scale

g. Cognitive performance in a range of executive functions including attention, inhibition, and timing

2. Show sustained effects at 6 months in all primary and secondary outcome measures.

3. Have a good safety profile measured weekly during the trial and at follow-up in questionnaires of side effects adapted to eTNS rated by children and parents and adverse events recording in the adverse events log.

4. Affect the sleep patterns of children with ADHD rated by parents using the parent-reported Sleep Disturbance Scale for Children (SDSC).

5. Improve physiological measures:

a) Heart rate; heart rate variability and objective hyperactivity measures tested in a wrist-hand device measured at baseline and post-treatment

b) Pupil diameter and head movement measured during rest and one of the cognitive tasks measured at baseline and post-treatment and at follow-up

6. Mechanistic objective:

To assess the mechanisms of action of eTNS on brain activation in ADHD children between 10- and 18-years using fMRI during 3 tasks and a resting state measured at pre and post treatment timepoints.

**2.3 Endpoints**

**The primary endpoint** will be the Investigator-scored parent-rated ADHD symptoms measured on the well validated ADHD-RS at baseline and weekly during the 4 week trial . The ADHD-RS recorded at the 6 months follow up visit will be a secondary endpoint. The clinician/investigator scored parent-rated ADHD-RS is the most commonly used primary outcome measure in treatment trials of ADHD . The post-doc RAs will be trained in obtaining this measure by the clinicians.

**Secondary endpoints:**

1. The teacher-rated ADHD symptoms assessed in the ADHD-RS (10min)
2. Teacher rated severity of ADHD symptoms using the Conners Teacher Rating Scale short form T-S (10 min)
3. Self-reported outcome measure in the Strength and Difficulties Questionnaire (SDQ) (10min)
4. A scale that measures emotional dysregulation, the parent (ARI-P) and child (ARI-S) rated Irritability questionnaire (Affective reactivity Index) (2min)
5. A questionnaire that measures the degree of mind-wandering rated by children, the Mind Excessive Wandering Scale (MEWS)(5min)
6. Measures of depression and anxiety assessed in the child and parent rated short forms of the revised Child and Adolescent Depression scale (RCADS-25 and RCADS-25-P) (15min)
7. Measures of the Columba Suicide Severity Rating Scale (2 min) (C-SSRS)
8. Parent-reported sleep quality measured in the parent-reported Sleep Disturbance Scale for Children (SDSC) (10min)
9. Executive functions: performance on a cognitive task battery developed for ADHD including several executive functions using the following measures: (One hour)
   1. Percentage of Omission and Commissions errors in the CPT sustained attention task
   2. Percentage of Omission and Commission errors in the vigilance task
   3. Probability of inhibition in the GNG task
   4. Simon RT effect for the Simon interference inhibition task
   5. Percentage of errors in the time estimation task
   6. Composite measures of mean reaction time, coefficient of variation of reaction time and percentage of premature errors across the GNG, CPT and Simon tasks
10. Physiological measures: This measure will only be measured before and after treatment. The specific measures will be:
    1. Heart rate, heart rate variability and objective hyperactivity measures will be measured in a wrist-held electronic device for the time of testing (3-4 hours each session) (Electrodermal Activity, Heart Rate Variability, Movement Variability)
    2. Pupil diameter (and head movement (coordinates of eye position)) will be measured at rest for 1 min and during one of the computer tasks
11. Safety measures: Safety will be assessed through weekly parent and children completed side effects rating scales adapted for eTNS . (3 min). In addition, there will be open questions, asking about general adverse events and their severity during the study period which will be recorded on the adverse events log and reported if needed.
12. Mechanistic objective: To assess the mechanisms of action of eTNS on brain activation in ADHD children between 10- and 18-years using fMRI during 3 tasks and a resting state measured at pre and post treatment timepoints.

**2.4 Trial design**

UK, multicentre, phase IIb, double-blind (participant, parent, researcher, and analyst), parallel group, sham-controlled, superiority randomised controlled trial. Participants will be randomised to real eTNS or sham eTNS.

The study will be conducted across two centres to increase representativeness and higher rates of participant enrolment. The treatment duration will be 4 weeks as this has been shown to be the optimal time period to elicit changes in ADHD symptoms in the pilot and pivotal studies. This is based on findings from the open 8-week trial where clinical changes were apparent in the first 4 weeks to avoid drop-outs in longer sham-controlled trials. A follow-up period of 6 months was chosen as this is standard for longer-term effects in studies of neurotherapies (and drugs) in ADHD.

**2.5 Method of allocation of groups**

King’s Clinical Trials Unit (KCTU) will generate the allocation 1:1 by minimisation by sex, medication, site, and age using an online web based KCTU randomisation management system for the duration of the project. A web-based randomisation system will be designed, using the bespoke KCTU randomisation system. The randomisation system was created in collaboration with the trial analyst/s and the CI and maintained by the King’s Clinical Trials Unit for the duration of the project. It is hosted on a dedicated server within KCL.

**2.6 Duration of the treatment period**

The intervention will be applied every night for 8 hours over 4 weeks. Participants will be tested for eligibility, pre-assessed, then receive the intervention for 4 weeks which they will apply themselves at home, receive a post-assessment a few days (+7 days) after treatment end, and will be assessed at follow-up at 6 months after randomisation (+- 6 weeks). The duration of the intervention is 4 weeks. The total length of participant participation in the trial (including eligibility, pre- and post-assessments, and follow-up assessments) is therefore about 6 months and a few days/weeks.

**2.7 Frequency and duration of follow-up**

The primary outcome will be collected at baseline, at 1, 2, 3, and 4 weeks and at 6 months (post-randomisation). Secondary outcomes will be collected at baseline, 4 weeks, and 6 months only.

**2.8 Visit windows**

Visits window for the 4 week assessment is +7 days, and +/- 6 weeks for the 6 months follow up visit.

**2.9 Data collection**

Please see the protocol for a detailed explanation of data collection.

**2.10 Inclusion/Exclusion criteria**

***Inclusion criteria***

1) Children and adolescents, aged 8-18 years at study entry

2) ADHD diagnosis (DSM-5; based on the K-SADS)

3) A score higher than 24 on the investigator-scored parent-rated ADHD-RS (DSM-5) (to include participants who still have relatively high symptoms)

4) Scoring above clinical cut-off for ADHD (5 or above) on the combined summary score of the child and adult ratings Kiddie Schedule for Affective Disorders and Schizophrenia, for School-age Children- present and lifetime version, ADHD module (K-SADS)

5) Both parent and child need to speak English (defined as sufficient to complete study assessments)

6) IQ above 70 as assessed on the Wechsler Abbreviated Scale of Intelligence (WASI-II) (to exclude participants with learning disability)

7) Patients should be either medication-naïve, OR willing to come off their stimulant medication for one week before the trial OR willing to be on stable medication for the duration of the trial.

***Exclusion criteria***

1) Comorbidity with any other major psychiatric disorder (except conduct/oppositional defiant disorder, mild anxiety, and depression- as assessed on the K-SADS, as these are commonly associated with ADHD)

2) Alcohol and/ or substance abuse (as assessed on the K-SADS) (potential confound)

3) Neurological abnormalities, such as epilepsy (potential confound)

4) Current medication with atomoxetine or guanfacine in the past two weeks (as these have an effect on the arousal system to be improved with eTNS)

5) Participants who usually take drug holidays on weekends or holidays will not be able to participate in the study unless they are willing to take their stimulant medication in a stable way throughout the study or not at all throughout the study and 1 week before the study (Participants will be either on medication or off medication to decrease heterogeneity)

6) Implanted cardiac or neurostimulation systems (contraindication to eTNS)

7) Implanted metallic or electronic device in their head (contraindication to eTNS)

8) Presence of body worn devices (e.g., insulin pumps and t-VNS) (contraindication to eTNS)

9) Currently receiving any non-medical treatment (e.g., psychotherapy, counselling, parent-training, cognitive rehabilitation, EEG neurofeedback) (potential confound)

10) Participants with dermatitis (could be sensitive to patches)

11) Traumatic Brain Injury (TBI) (potential confound)

Additional Exclusion Criteria for the 56 patients that will participate in the fMRI study

12) Under 10 years old

13) Have any MRI contra-indications (e.g., metal implants, pacemakers, braces, tattoos/piercings claustrophobia etc.) which would render them unsuitable for the fMRI sub-study

14) Be pregnant and/or breastfeeding if female

If patients are COVID positive, the participant’s involvement in the trial will be delayed. If any patient develops COVID during the trial, arrangements will be made as required for the individual case.

**2.11 Measures**

A detailed description of data collected will be given in the Schedule of Assessments and Measures (Section 7 of this document). Scoring for the primary and secondary outcomes will be provided in Section 8.

### 2.11.1 Measures collected at Baseline only

- Demographics: child age, child sex, child ethnicity, parent/guardian sex, parent/guardian relationship to child, socioeconomic status/deprivation (as measured by parent education, income, and indices of multiple deprivation)
- Background information; child age at ADHD diagnosis, use of previous ADHD medication
- Comorbid affective disorders as defined by the Kiddie Schedule for Affective Disorders and Schizophrenia

**2.11.2 Primary outcome/endpoint**

Investigator-assessed parent ratings of the ADHD-RS at 4 weeks.

**2.11.3 Secondary outcomes**

The following secondary outcome measures will be covered by this Statistical Analysis Plan and form part of the Primary outcome paper:

1. Investigator-assessed parent ratings of the ADHD-RS at 6 months (total score)

The following outcomes at 4 weeks and at 6 months:

1. Child-rated Strength and Difficulties Questionnaire (SDQ) ADHD subscore
2. Teacher-rated severity of ADHD symptoms as assessed by the Conners Teacher Rating Scale
3. Teacher rated ADHD-RS total score
4. Parent rated Irritability questionnaire total score (ARI-P)
5. Child rated Irritability questionnaire total score (ARI-S)
6. Self-rated Mind Excessive Wandering Questionnaire (MEWS) total score
7. Revised child and adolescent depression scale rated by children (RCADS-25)
8. Revised child and adolescent depression scale rated by parents (RCADS 25-P)
9. Columbia Suicide Severity Scale – Suicidal Ideation Score (child rated)
10. Cognitive performance - Vigilance Task (percentage of Omission errors)
11. Cognitive performance - Vigilance Task (percentage of Commission errors)
12. Parent-reported Sleep Disturbance Scale for Children total score (SDSC)
13. Objective hyperactivity (at 4 weeks only). This will be measured as a composite score of intensity of movement and frequency of movement.
14. Pupil diameter
15. Side effects score (rated by child)
16. Side effects score (rated by parent)
17. Weight
18. Pulse

Safety will also be assessed and reported using the Adverse Events log and the Side Effects Scale (Child and Parent). See Section 4.6.

Teacher rated measures (3 & 4) will not be analysed if these measures are missing for >=60% of randomised participants or if the completion rates are differential by arm (>15% difference). In these scenario, these measures will only be reported descriptively.

**The following outcome measures will be exploratory and not necessarily form part of the Primary outcome paper and are not covered by this analysis plan:**

- Cognitive performance - Go-No Go Task (Probability of inhibition)
- Cognitive performance - Continuous Performance Task (percentage of Omission errors)
- Cognitive performance - Continuous Performance Task (percentage of Commission errors)
- Cognitive performance - Interference Inhibition (Simon Reaction time (RT) effect)
- Cognitive performance - Time Estimation Task (Percentage errors)
- Cognitive performance - Composite measure MRT (mean reaction time)
- Cognitive performance - Composite measure CV (coefficient of variance)
- Cognitive performance – Composite percentage of premature responses
- Heart rate (at 4 weeks only)
- Heart rate variability (at 4 weeks only)
- Head movement

**The following outcome measures will be mechanistic and not necessarily form part of the Primary outcome paper and are not covered by this analysis plan:**

- Mechanisms of action of eTNS on brain activation in ADHD children between 10- and 18-years using fMRI during 3 tasks and a resting state measured at pre and post treatment timepoints

**2.12 Sample size estimation (including clinical significance)**

Based on the previous trial, we anticipate a between group reduction in ADHD symptoms at 4 weeks between real eTNS versus sham with an effect size (Cohen’s d) = 0.5 (McGough et al., 2019 (3)). Using a baseline to post-treatment correlation = 0.5, with 90% power, and a 5% type I error, we estimate that we will need to recruit 128 participants (64:64). In order to account for a loss to follow up rate of 15% (which is a conservative estimate given our attrition rate of 9% for our fMRI Neurofeedback trial and of 0% for our tDCS trial), we have inflated the number randomised to 150 (75:75).

The sample size has been checked following KCTU-Statistics Standard Operating Procedures ST-05 and stored as signed as an essential document.

**3. DATA ANALYSIS PLAN – DESCRIPTIVES**

**3.1 Recruitment and representativeness of recruited patients**

A CONSORT flow chart will be constructed (2) – see Figure 1. This will include the number of eligible patients, number of patients consenting to enter the trial, number of patients refusing, then by treatment arm: the number continuing through the trial, the number withdrawing, the number lost to follow-up and the numbers excluded/analysed

Figure 1 – Template CONSORT diagram

**3.2** **Baseline comparability of randomised groups**

Baseline descriptions of participants by treatment and overall: means and standard deviation, medians and upper and lower quartiles, or numbers and proportions will be reported as appropriate.

No significance testing will be used to test baseline difference between the randomised treatment groups.

The following baseline variables will be described both overall and between randomised groups:

- Demographics: child age, child sex, child ethnicity, parent/guardian sex, parent/guardian relationship to child, socioeconomic status/deprivation (as measured by parent education, income, house ownership, number of people living in the house, number of bedrooms and indices of multiple deprivation)
- Stimulant medication status (On stable medication, Off medication/naive)
- Background information; child age at ADHD diagnosis, use of previous ADHD medication
- Child IQ (WASI-II score)
- Handedness (Edinburgh Handedness Inventory – Short Form)
- Percentage who have comorbid conduct and oppositional defiant disorders as defined by the Kiddie Schedule for Affective Disorders and Schizophrenia
- ADHD research diagnosis (combined presentation, inattentive presentation, hyperactive/impulsive presentation) as defined by the Kiddie Schedule for Affective Disorders and Schizophrenia

**3.3 Anticipated intercurrent events**

Discontinuation of intervention (sham or real TNS) estimated rate <10%

We have not considered Death of child or guardian to be an anticipated intercurrent event as we expect rates of these events to be negligible.

Withdrawal from data collection (withdrawal of consent, uncontactable or unable to travel to assessment) also affects the outcomes as later measurements will be missing, but this affects the analysis and not the estimand so is not considered an intercurrent event.

**3.4 Adherence to allocated treatment and treatment fidelity**

We will ask participants to record adherence to the study and to record the length of time eTNS was applied each night in a daily sleep diary. We will emphasize adherence and the importance to honestly report usage of the device.

Adherence for the purposes of analysis will be defined as a binary variable; having used the device for 60% of nights over the 4 week treatment period (therefore using it for at least 17 nights out of 28 nights), with “using the device for a night” defined as using it for at least 1 hour in a night.

**3.5 Loss to follow-up and other missing data**

It is the aim of the trial to minimise withdrawal of participants from treatment and follow-up. Completion of a withdrawal from trial is regarded as withdrawal from data collection/follow-up in this analysis; participants who discontinue the intervention will still be followed up where possible.

Withdrawal from trial will be reported by intervention group. Moreover, the proportions of participants missing each variable will be summarised in each arm and at each time point. The numbers, proportions, and reasons for withdrawal from trial will be summarised by treatment arm. The distribution of times between randomisation and withdrawal from follow-up will be summarised using a histogram.

The baseline characteristics of those missing follow-up will be compared to those with complete follow-up (using list as per Section 3.2). Data will be explored for pattern missingness.

**3.6 Adverse event reporting / Reporting of side effects**

Adverse events (AE), adverse reactions/adverse device effects (AR/ADEs), serious adverse events (SAE) and serious adverse reactions/adverse device effects (SAR/SADEs) will be summarised by type (listed below as defined in the eCRF), trial arm and time point.

Adverse device effects are any adverse events considered related to the investigational medical device. Theoretically, adverse events and serious adverse events could occur that are judged not to be related to the device but are judged to be related to the trial procedures (“any procedure specific to the clinical investigation”). These will be classified as adverse reactions as opposed to adverse device effects.

For adverse reactions/adverse device effects, Relatedness is collected as ‘Casual, Probable, Possible, Unlikely and Not related’ where ‘causal, probably and possible’ are considered related and ‘unlikely or none’ are not considered related.

Adverse events will be reported cumulatively at each follow up timepoint. Additionally, risk ratios for adverse events by type will be reported (using all participants’ reported AEs post-randomisation i.e., the ITT population) with 95% confidence intervals. These will be reported graphically in a dotplot in approach as proposed by Amit, Heiberger, and Lane (2008) (4) and implemented in Stata in the aedot package (5).

Types of adverse events:

1. Anxiety or worrying

2. Tearfulness, sadness, or depression

3. Tiredness, demotivation, joylessness

4. Withdrawal or less socializing (less interested in peers)

5. Grumpiness and irritability

6. Twitching or ticks (eye blinking, head tics)

7. Scratching himself/ herself, biting nails or lips more

8. Headaches

9. Stomach aches

10. Lack of appetite

11. Problems falling asleep or sleep problems

12. Frustration

13. Lack of confidence

14. Distractibility

15. Other physiological event

16. Other psychological event

Other physiological events and other psychological events will be further broken down by system organ class (see Table 9 in Section 9).

Additionally, side effects reported in the side effects scale (which may overlap with adverse events) will be described. Rates of side effects will be summarised over the 4 week treatment period. Changes in the top most reported side effects over time will also be described. Side effects reported by the participants and by parents will be described separately.

## **3.7 Assessment of outcome measures (planned unblinding of trial statistician)**

Planned unblinding is based upon KCTU-Statistics SOP ST:06 Protocol Review and the protocol. All members of the research team will be blinded except the trial manager and the trial statistician who may be unblinded following approval of the first version of the SAP. The senior statistician will remain (fully) blind.

The DMEC will not see accumulating data by randomised group initially. All routine DMEC reports will be aggregated across treatment group unless there is a substantial safety concern (for example once a minimum of 5 SAEs and SADEs have been reported).

If there is a substantial concern of safety is raised the DMEC may request a closed report, unblinded to treatment allocation. However, this will not be prior to a minimum number of patients are recruited and followed up to provide the DMEC with adequate information to reassure their concerns.

## **3.8 Blinding status of personnel and planned unblinding**

All participants, Chief Investigator, Site Principal Investigators, researchers, and outcome assessors will be fully blinded throughout the trial. The trial manager will be unblinded to a patients’ treatment allocation to allow allocation of the correct device to each participant.

The Trial Statistician will be fully blind until at least the first version of the Statistical Analysis Plan (SAP) is approved by the Trial Steering Committee (TSC) as per KCTU SOP ST-02 (Statistical Analysis Plan). After the first version of the SAP is approved by the TSC, the Trial Statistician may become fully unblinded if required for the DMEC meetings (see Section 3.8) Otherwise, the Trial Statistician will be unblinded until the point of the final analysis.

As per KCTU SOP ST-06, the Senior Statistician (and all other researchers) will be fully blind to treatment allocation until after database lock at completion of the final analysis. Descriptive statistics for outcome measures.

Each of the outcome measures will be described by treatment group. Means, standard deviations, or medians and interquartile ranges will be used for continuous variables. Frequencies and proportions will be used to describe categorical variables. Events will be described as the number occurred by each timepoint.

## **3.9** **Listing of concomitant medications**

The percentage of participants who are taking each type of medication (Stimulant Medication, Psychotropic medication and Other) will be described at baseline, 4 weeks, and 6 months by trial arm.

## **3.10 Other descriptives (blinding/acceptability)**

Guesses of which intervention (Real or Sham eTNS) they received will be described after the 1^st^ and 4th week intervention period by the child, parent and by the researcher.

Acceptability will also be described from the acceptability survey at week 4

## **4. DATA ANALYSIS PLAN – INFERENTIAL ANALYSE****S**

## **4.1 Primary analysis description**

### 4.1.1 Definition of primary estimand

The primary clinical question of interest is what is the difference in investigator-scored parent-rated ADHD-RS score at 4 weeks post-randomisation for youth with ADHD offered real eTNS compared to sham eTNS (every night for 4 weeks), regardless of discontinuation of the intervention (as described below)

The estimand is described by the following attributes:

- **Population**: Children and adolescents aged 8-18 years with a clinical or research ADHD diagnosis (and who have relatively high symptoms – 24 or higher on the parent rated ADHD-RS)
- **Endpoint**: ADHD-RS score at 4 weeks post-randomisation
- **Treatment condition**: real eTNS compared to sham eTNS regardless of discontinuation for any reason (treatment policy strategy)
- **Remaining intercurrent events:** The intercurrent event “intervention discontinuation” is already addressed by the treatment condition of interest attribute. There are no other expected intercurrent events
- **Population-level summary:** The (adjusted) mean difference

**Rationale for estimand**: To compare the offer of 4 weeks of real eTNS to sham eTNS.

### 4.1.2 Further details on handling of intercurrent events and subsequent supplementary analyses

As stated, for the primary analysis, a treatment policy strategy will be used for the ICE (intercurrent event) of discontinuation of treatment; that is the occurrence of the intercurrent event will be considered irrelevant in defining the treatment effect of interest and the value for the variable of interest will be used regardless of whether or not the intercurrent event occurs. A separate supplementary analysis using a different strategy will be covered as per Section 4.3 and Section 4.13.

No other intercurrent events are expected, however if additional potential intercurrent events are determined throughout the trial at a rate >5%, additional sensitivity analyses will be considered by the blinded analyst (senior statistician) and may be added to this SAP or listed/considered as post-hoc.

## **4.2 Main analysis of treatment differences (intention-to-treat population set)**

The main analysis will include all randomised participants (eligibility as defined in section 2.9) in the arms to which they were randomised, regardless of what treatment they received post-randomisation, with the intent to estimate an intention-to-treat (ITT) type estimand. This will be estimated for the primary outcome and all secondary outcomes.

## **4.3 Per protocol (complier average causal effect) analysis**

As per Section 5.1, the aim of the main analysis is to estimate an Intention to treat (ITT) type estimand. A separate analysis will also be carried out to estimate efficacy of the intervention in compliers (a complier average causal effect). The per-protocol population will be the same as the ITT population except including only participants who adhered to the treatment schedule (as defined in Section 3.4) and excluding other protocol violators. This will form the principal strata of compliers for the purpose of the complier average causal effect (CACE) analysis (which will be described in Section 4.13) Protocol deviators as defined below will not be excluded.

**4.3.1 Definition of protocol deviation/violation**

A protocol deviation/violation is defined as any excursion from the protocol. Examples of possible protocol deviations/violations include participants who do not meet the eligibility criteria and are randomised in error and participant assessments occurring outside the specified visit windows.

A protocol deviation (PD) is defined as a non-serious breach from the protocol that is unlikely to lead to any impact on the value of the data contributing to the overall treatment effect. A protocol violation (PV) is an excursion from the protocol that is likely to lead to an impact on the estimate of the “efficacy” of the intervention. Protocol deviation and violations will be reported (and tabulated by arm). Protocol deviators and protocol violators are participants that experience a protocol deviation or protocol violation, respectively.

All visit window deviations will be classified as protocol deviations rather than protocol violations as the primary analysis uses actual time since randomisation and therefore deviations from the visit windows are not impactful for this analysis. An assessment of the impact of other excursions from the protocol on the primary outcome will be made by the Chief investigator and Senior statistician. All protocol violations will be reported to the DMEC as well as any excursions for which the trial team are unsure if should be considered deviations or violations (for which the DMEC will have the final decision on).

**4.4 Analysis of primary outcomes**

**4.4.1 Primary Efficacy Parameters**

The primary analysis will be analysed using a mixed-effects linear model, fitting 4-week ADHD symptoms scores, each patient will be fitted as a random intercept, and adjusted for fixed effects of baseline ADHD score, site, age (8-13.5, 13.6-19), gender (Male, Female), and medication (On Medication, Off Medication/Medication naïve).

Time will be included as a continuous covariate using actual time of measurements post-randomisation for the week 1-4 timepoints, to model a linear effect over time. A time by treatment interaction will allow post-estimation calculation of treatment effects at the specific timepoints, as well as an assessment of heterogeneity of the treatment effect over time. A random slope will be included to account for subject-specific variation in the time trend. The linear assumption will be examined and if this appears to be violated, timepoint will be included instead of actual time, as a dummy coded categorical variable, assuming no particular shape for the effect over time.

Treatment differences at the Month 6 timepoint are not expected to follow the same linear time trend and so a separate model will be fit for this analysis, including all follow up timepoints and using the same approach as above except that time will be included as dummy coded categorical variable allowing treatment effects to be independent by timepoint. A random slope will not be included in this model.

95% confidence intervals, p-values, and effect sizes (standardised mean differences calculated using baseline pooled standard deviation estimate) will be estimated for between group differences with alpha=0.05.

**4.5 Analysis of secondary outcomes**

All secondary analyses will use a significance level=0.05.

All secondary outcomes are measured as continuous (with some measured as percentages).

These will be analysed with (longitudinal) multi-level mixed-effects multivariable linear regression models using all follow up time-points. Secondary outcomes are recorded at the week 4 and 6 month timepoints only (and not at week 1 – week 3). As such time will be included as categorical, making no assumption of a linear effect of treatment over time. Post-estimation commands will be used to get estimates at specific follow up times. A random intercept will be fitted by participant and the analysis adjusted by terms consistent with the primary outcome as well as baseline values of the outcome where appropriate. Unadjusted and adjusted results will be presented.

Outcomes measured as percentages will be modelled in the same way, unless there are a large number of values at the bounds (0 or 100) or the residuals otherwise appear skewed. In this scenario, other strategies will be considered such as data transformation prior to analysis.

**4.6 Safety**

No additional safety outcomes will be analysed other than covered by existing secondary outcomes or descriptives of AEs (risk ratios by system body class) as in Section 3.6.

**4.7 Time points**

As per Section 2.8, visit windows exist for all follow up visits. However, all available data will be used for the main analyses regardless of compliance to visit windows.

**4.8 Stratification and clustering**

Randomisation has been stratified by site, gender, medication, and age. Therefore, it is important to include these variables in the modelling process; they will be adjusted for as fixed effects.

The structure of primary outcome (and some of the secondary outcome) data is longitudinal with repeated measurements. This correlation of observations within participants will be accounted for in the modelling process by including a subject-varying random intercept.

**4.9 Missing items in scales and subscales**

The number (%) with complete data will be reported. If missing data guidance is provided for missing items within the scale scoring guide, this will be used (see Appendix X) If no guidance is provided scales will be pro-rated for an individual if 20% or fewer items are missing. For example, in a scale with 10 items, prorating will be applied to individuals with 1 or 2 items missing. The average value for the 8 or 9 complete items will be calculated for that individual and used to replace the missing values. The scale score will be calculated based on the complete values and these replacements.

**4.10 Missing baseline data**

If there is missing baseline data for an outcome, an approach such as including a missing indicator for baseline as a covariate in the model may be used as per the recommendations of White and Thompson (6).

**4.11 Missing outcome data**

The mixed model will be fitted using maximum likelihood methods that are valid under the MAR assumption. However, this assumption relates to the variables that are included in the model. To allow for a variable which predicts missingness, this variable needs to be included as either one of the explanatory or dependent variables in the model. Demographic or clinical baseline variables (those listed in Section 3.2) will be investigated as predictors of missingness. Should any of these variables be predictors of outcome missingness then such variables will be included as covariates in the model if deemed suitable for adjustment.

If post treatment variables such as compliance with treatment, or baseline variables not suitable to adjust for in the main analyses, are found to be predictive of drop out, inverse probability weighting will be considered to account for these variables.

**4.12 Method for handling multiple comparisons**

We have specified a single primary outcome (and endpoint) and all other secondary analyses will be considered exploratory and subsidiary. As such no adjustment will be considered for multiple comparisons (7), allowing reviewers to make their own adjustment to estimates if they wish.

**4.12 Method for handling non-compliance**

Compliance and adherence (as defined in Section 3.4) will be summarised.

As per Section 4.3, we will estimate the average treatment effect for the subpopulation of participants who adhered to the intervention using a complier average causal effect (CACE) estimand, with other protocol violators also defined as not compliers. If there are a non-negligible number of protocol violators who did adhere to the intervention (as defined above), a separate CACE analysis will also be carried out that does not exclude these participants.

We will not account for patterns of compliance (i.e., compliance over time) in such an analysis unless the data suggests large variation in such patterns. If there is sufficient variance in compliance (measured continuously by number of nights used the device), exploratory analyses examining the dose-response relationship will be carried out.

**4.13 Model assumption checks**

The models assume normally distributed error terms (residuals); these will be checked graphically following model fit and if these appear non-normal, transformations or bootstrapping of the standard errors (and subsequent confidence intervals) will be considered.

**4.15 Sensitivity analyses**

The following sensitivity analyses will be carried out for the primary analysis:

The impact of departures from the missing at random (MAR) assumption on treatment effects will be assessed using pattern mixture models (8) to give results for a range of scenarios for the possible unobserved values

Some number of participants randomised may be siblings. If >20% of the randomised participants are siblings, we will carry out a sensitivity analysis to examine the effect of clustering by sibling pairs. We will aim to do this by including an additional sibling-pair random intercept in the primary analysis model.

**4.16 Planned subgroup analyses**

Evidence in differences in treatment effect by subgroups will be assessed by tests of interaction; the primary analysis model will be used with an interaction term (trial arm*subgroup). Only if these tests show evidence of an interaction will treatment effects within these subgroups be presented.

All planned subgroup analyses are for the primary outcome only.

The following subgroup analysis will be carried out for the main paper:

Split by stimulant medication status (On stable medication, Off medication/naive)

**5. QUALITY CONTROL AND QUALITY ASSURANCE**

This trial is conducted by KCTU using the KCTU Quality Management systems including Incident reporting, KCTU SOPs and the Statistics File Upload System for storing essential statistical documents. Quality control and assurance is maintained following KCTU-Statistics ST:04 Statistical Quality Assurance.

**5.1 Quality control**

The junior statistician will carry out data checks on MACRO data prior to preparing DMEC reports (as per KCTU Statistical SOP ST-01) and prior to final data lock. Generated data queries will be provided to the trial management team in an Excel file to be communicated to sites. Responses should be sent back to the statistician and unresolved data queries will be re-issued. Where possible data queries should be resolved prior to DMEC reports/interim analyses, where this is not possible, outstanding data issues will be made clear to the DMEC on the report. Syntax for data checking will be written in accordance with KCTU Statistical SOP ST-08 (Data manipulation) to allow reproducibility and for purposes of an audit trail. All syntax, output, resulting queries and responses from sites (via the trial management team) will be retained as per KCTU Statistical SOP ST-03 (Statistical Document Retention).

**5.2 Carrying out the final analysis**

For the final analysis, the Senior TS will review the code (that will be written in such a way as to not unblind) but will not see results until finalisation of the analysis.

As per KCTU Statistical SOP ST-08, all syntax will be written in such a way to allow for the analysis to be reproduced for purposes of an audit trail, and syntax will be appropriately commented to allow interpretation and for reproducibility. All syntax and output will be retained on local filing systems and uploaded to the Statistics File Upload System (as per KCTU Statistical SOP ST-03 (Statistical Document Retention)).

**5.3 Approval and version control of the SAP**

As per KCTU Statistical SOP ST-02 Statistical Analysis Plan, after initial sign off of the SAP, if any changes are required, these will be decided by the blinded senior TS and not by the unblinded trial statistician, so as to avoid any chance or appearance of introducing bias having seen unblinded data. A copy of the tracked changes version will be retained, and the updated version will be approved by the Chief Investigator, Senior Statistician and TSC chair. Any changes between versions will also be outlined in Section 12.

The initial signed off version will be versioned as Version 1.0. Minor amendments following this should be versioned with sub-numbering e.g., Version 1.1, Version 1.2 etc. Major amendments should be numbered as Version 2.0, 3.0 etc.

**5.4 Quality Assurance**

Quality assurance processes will be followed according to KCTU Statistical SOP ST-04 Statistical QA.

**6. SOFTWARE**

Data management: An online data collection system for clinical trials (MACRO; Elsevier) will be used. This is hosted on a dedicated server at KCL and managed by the King’s CTU. The KCTU will extract data periodically as needed and provide these in comma separated (.csv) or Stata (.dta) format.

Statistical analysis: Stata (version 17 or higher) will be used for the main analyses. R (version 3.6.3 or higher) and R markdown may also be used for producing graphs and tables and automating report writing.

# 7. SCHEDULE OF ASSESSMENTS AND MEASURES

Data being collected from patients and recorded in MACRO:

|  | | Eligibility | Baseline | After randomisation | eTNS  1^st^ week | eTNS  2^nd^ week | eTNS  3^rd^ week | eTNS  4^th^ week | 6-month after randomisation |
| --- | --- | --- | --- | --- | --- | --- | --- | --- | --- |
| Consent form child (2 min) | | x | x |  |  |  |  |  |  |
| Consent form parent (2 min) | | x | x |  |  |  |  |  |  |
| Eligibility checklist (10 min) | | x |  |  |  |  |  |  |  |
| Background information (10 min) | | x |  |  |  |  |  |  |  |
| Kiddie Schedule for Affective Disorders and Schizophrenia (K-SADS) (1-1.30 hr) (P) | | x |  |  |  |  |  |  |  |
| Kiddie Schedule for Affective Disorders and Schizophrenia (K-SADS) (50 min) (C) | | x |  |  |  |  |  |  |  |
| ADHD Rating Scale (ADHD-RS) (P) (10 min) | | x | x |  | x | x | x | x | x |
| WASI-II (40 min) | | x |  |  |  |  |  |  |  |
| ADHD Rating Scale (ADHD-RS) (T) (10 min) | |  | x |  |  |  |  | x | x |
| Conners’ Teacher Rating Scale (Conners 3 T--S) (T) (10 min) | |  | x |  |  |  |  | x | x |
| Strength and Difficulties Questionnaire (SDQ) (C) (10 min) | |  | X |  |  |  |  | X | X |
| Edinburgh Handedness Inventory-short form (C) (1 min) | | x |  |  |  |  |  |  |  |
| Columbia Suicide Severity Rating Scale (C-SSRS) (Child) (2 min) | |  | x |  |  |  |  | x | x |
| Affective Reactivity Index self-rating (ARI-S) (C) (2 min) | |  | x |  |  |  |  | x | x |
| Affective Reactivity Index self-rating (ARI-P) (P) (2 min) | |  | x |  |  |  |  | x | x |
| Mind Excessively Wandering Scale (MEWS) (C) (5 min) | |  | x |  |  |  |  | x | x |
| Revised Children’s Anxiety and Depression Scale (RCADS-25) (P) (15 min) | |  | x |  |  |  |  | x | x |
| Revised Children’s Anxiety and Depression Scale (RCADS-25) (C) (15 min) | |  | x |  |  |  |  | x | x |
| Sleep disturbance Scale for Children (SDSC) (P) (10 min) | |  | x |  |  |  |  | x | x |
| Sleep diary (2 min) | |  |  |  | x | x | x | x |  |
| Blinding Questionnaire parents/ child/ researcher (2min) | |  |  |  | x |  |  | x |  |
| Acceptability survey (P) (C) (2 min) | |  |  |  |  |  |  | x |  |
|  | **Device training** | | | | | | | | |
| Parents trained in device use (20-30 min) | |  |  | x |  |  |  |  |  |
| Use of device | |  |  |  | x | x | x | x |  |
| Trial Manager to check device use with family during week 1 | |  |  |  | x |  |  |  |  |
|  | **Safety measures** | | | | | | | | |
| Side effects (3 min) (C, P) | |  | x |  | x | x | x | x | x |
| Adverse events log (3 min) (C, P) | |  |  |  | x | x | x | x | x |
| Concomitant medication log (3 min) | |  | x |  | x | x | x | x | x |
|  | **Lab measures for monitoring** | | | | | | | | |
| Height, weight, and vital signs (10 min) | |  | x |  |  |  |  | x | x |
|  | **Neurocognitive** **measures** | | | | | | | | |
| Go-no go task (5 min) | |  | x |  |  |  |  | x | x |
| Continuous performance task (8 min) | |  | x |  |  |  |  | x | x |
| Interference inhibition (Simon Task) (5 min) | |  | x |  |  |  |  | x | x |
| Time estimation task (5 min) | |  | x |  |  |  |  | x | x |
| Vigilance task (5 min) | |  | x |  |  |  |  | x | x |
|  | **Physiological measures** | | | | | | | | |
| Objective measure of heart rate, its variability and hyperactivity measure (wrist-held electronic device) to be used for 3-4 hours during the pre and post treatment research visits | |  | x |  |  |  |  | x |  |
| Pupil diameter and head motion during rest and tasks | |  | x |  |  |  |  | x | x |
|  | **fMRI measures in subgroup of 56 patients** | | | | | | | | |
| Mock scan (can only take place at the research centre) | |  | x |  |  |  |  |  |  |
| fMRI safety form (5 min) | |  | x |  |  |  |  | x |  |
| fMRI request form (10 min) | |  | x |  |  |  |  |  |  |
| Stop task (6 min) | |  | x |  |  |  |  | x |  |
| Sustained Attention task (12 min) | |  | x |  |  |  |  | x |  |
| Working memory task (5 min) | |  | x |  |  |  |  | x |  |
| Resting state fMRI (10 min) | |  | x |  |  |  |  | x |  |
|  | **Forms** | | | | | | | | |
| Receipt of payment (1 min) | | x | x |  |  |  |  | x | x |

# 8. SCORING

| **Outcome** | **Title** | **Scoring present?** | **Timescale** | **Items** | **Scoring description** | **Missing items** | **Interpretation** |
| --- | --- | --- | --- | --- | --- | --- | --- |
| Primary outcome | ADHD Rating Scale (ADHD-RS) – Home Adolescent | YES | Over the past week | 18 broken up into 2 subscales, inattention (1-9) and hyperactivity (10-18).  Additional “How much do these behaviours cause problems” questions not included in scoring. | Total raw score – obtained by adding together the Inattention and Hyperactivity subscale raw scores. | No guidance | Higher scores more severe |
| Primary outcome | ADHD Rating Scale (ADHD-RS) – Home Child | YES |  |  |  |  |  |
| Secondary outcome | ADHD Rating Scale (ADHD -RS) – School Adolescent | YES |  |  |  |  |  |
| Secondary outcome | ADHD Rating Scale (ADHD-RS) – School Child | YES |  |  |  |  |  |
| Secondary outcome | Child-rated Strength and Difficulties Questionnaire (SDQ) ADHD subscore | YES | At visit- past month | 25 items divided into 5 subscales (emotional problems, conduct problems, hyperactivity, peer problems, prosocial) See “SDQEnglishUK4-17scoring-1” scoring document for items. Some items reversed.  Additional items not used in scoring | Hyperactivity/inattention score | Counted as missing if one of the 4 component scores is missing | Higher scores more severe |
| Secondary outcome | Conners’ Teacher Rating Scale (CTRS-R) | YES | past month | 39 items with raw score 0-3 | Raw scores converted into different scores as per Conners scoring grid and then summed into 7 domains. Overall score is a sum of the 7 domain scores. | No guidance | Higher scores more severe |
| Secondary outcome | ARI (Child) | Yes | At visit- last 7 days | 7 items scored 0,1 or 2. First 6 items used for scoring | Sum of the 6 items | No guidance | Higher scores more severe |
| Secondary outcome | ARI (Parent) | Yes | At visit-last 7 days | 7 items scored 0,1 or 2. First 6 items used for scoring | Sum of the 6 items | No guidance | Higher scores more severe |
| Secondary outcome | Mind Excessively Wandering Scale (MEWS) | Yes | At visit-last 7 days | 12 items scored 0-3 | Sum of the 12 items |  | Higher scores more severe |
| Secondary outcome | RCADS (Parent ) | Yes | At visit-last 7 days | 25 items scored 0-3 | Total Anxiety and Depression raw score (sum of all 25 items) | Mean replacement (pro-rating) if 5 or fewer items in the scale | Higher scores more severe |
| Secondary outcome | RCADS (Child) | Yes | At visit- last 7days | 25 items scored 0-3 | Total Anxiety and Depression raw score (sum of all 25 items) | Mean replacement (pro-rating) if 5 or fewer items in the scale | Higher scores more severe |
| Secondary outcome | Columbia Suicide Severity Scale (Screen version) | Yes | At visit- In past month | 7 Y/N questions | Suicide Ideation Score. Score 0-5 based on highest question answered Yes from Q1 – Q5 | No guidance | Higher scores more severe |
| Secondary outcome | Parent-reported Sleep Disturbance Scale for Children (SDSC) | Yes | At visit- 4 weeks | 6 factors - Disorders of initiating and maintaining sleep (sum items 1,2,3,4,5,10,11)  Sleep Breathing Disorders (sum items 13,14,15)  Disorders of arousal (sum items 17,20,21)  Sleep-Wake Transition Disorders (sum items 6,7,8,12,18,19)  Disorders of excessive somnolence (sum items 22,23,24,25,26)  Sleep Hyperhidrosis (sum the items 9,16) | Total score (sum 6 factors’ scores) | No guidance | Higher scores more severe |
| Secondary outcome | Go-No Go Task | Yes | At visit | Probability of inhibition | One item – percentage | NA | Higher score better performance |
| Secondary outcome | Continuous Performance Task | Yes | At visit | Omission errors (%), Commission errors (%) | Two items – separate outcomes (percentages) | NA | Higher score worse performance |
| Secondary outcome | Interference Inhibition (Simon Task) | Yes | At visit | Simon reaction time effect (Incongruent – congruent RT) | One item - continuous | NA | Higher score worse performance |
| Secondary outcome | Time Estimation Task | Yes | At visit | Errors (%) | One item – percentage | NA | Higher score worse performance |
| Secondary outcome | Vigilance Task | Yes | At visit | Omission errors (%), Commission errors (%) | Two items – separate outcomes (percentages) | NA | Higher score worse performance |
| Secondary outcome | Composite cognitive performance | Yes | At visit | Composite measure MRT (mean reaction time), Composite measure CV (coefficient of variance), Composite premature responses (%) | Three items – separate outcomes (2 continuous, 1 percentage) | NA | Higher score worse performance |
| Secondary outcome | Objective Hyperactivity Measure (movement) | Yes | At visit | Composite of frequency and intensity of movement | In the absence of previous evidence on how to weight the two components (as a measure of hyperactivity), the two components (frequency and intensity) will be given equal weighting. Each component will first be transformed into z scores and the two z scores will then be summed. | NA | Higher score worse |
| Secondary outcome | Pupil Diameter | Yes |  | At rest and during task, right and left eye | Two separate scores will be created and analysed, mean pupil diameter at rest (right eye + left eye/2) and mean pupil diameter during task (right eye + left eye/2) | No guidance | Higher score more arousal |
| Secondary outcome | Side effects Scale (2 measures, child and parent reported) | Yes | At visit- last 7 days | 37 separate side effects | For each of the 37 side effects, the worst reported level will be taken from over the 4 weeks of reporting (side effects reported at week 1,2,3 and 4). Each level is assigned a score from 0-3. The scores for each side effect will then be summed to get a total side effects score. Child and parent reported side effects will be reported separately. | NA | Higher scores more severe |
| Secondary outcome | Pulse | NA | At visit | NA | As recorded (beats per minute) | NA | higher or lower can be worse depends on age norms |
| Secondary outcome | Weight | NA | At visit | NA | As recorded (kg) | NA | higher or lower can be worse depends on age norms |

# 9. DUMMY TABLES FOR FINAL ANALYSIS

The below tables are examples only, may not be fully populated and are not exhaustive.

## 9.1 Descriptive baseline characteristics

Table 1 - Baseline characteristics/Demographics

| **Baseline characteristics (n,%)** | | **Real eTNS**  **N=xx** | **Sham eTNS**  **N=xx** |
| --- | --- | --- | --- |
| Child Age | Mean (SD), range |  |  |
| Child Sex at Birth | Female (n, %) |  |  |
|  | Male (n, %) |  |  |
| Child Ethnicity | White (n, %) |  |  |
|  | Mixed or Multiple ethnic groups (n, %) |  |  |
|  | Asian or Asian British (n, %) |  |  |
|  | Black, African, Caribbean, or Black British (n, %) |  |  |
|  | Other ethnic group (n, %) |  |  |
| Parent/Guardian Sex at Birth | Female (n, %) |  |  |
|  | Male (n, %) |  |  |
| Parent/Guardian Relationship to Child | Parent (n, %) |  |  |
|  | Stepparent (n, %) |  |  |
|  | Foster parent/guardian (n, %) |  |  |
|  | Grandparent (n, %) |  |  |
|  | Other relative (n, %) |  |  |
|  | Other non-relative (n, %) |  |  |
| Parent/guardian completed highest education Level | Primary School (SATS) (n, %) |  |  |
|  | Secondary School (GCSE) (n, %) |  |  |
|  | Further education (A-level/ IB/ NVQ/ BTEC) (n, %) |  |  |
|  | Higher Education (University) (Undergraduate degree) (n,%) |  |  |
|  | Postgraduate Education (Masters, PhD) |  |  |
| Indices of multiple deprivation (decile) | Mean (SD), range |  |  |
| Total Family income (gross income before taxes and other deductions) | None - on benefits |  |  |
|  | Up to £20,000 |  |  |
|  | Between £20,001- £35,000 |  |  |
|  | Between £35,001-£55,000 |  |  |
|  | Above £55,001 |  |  |
| Child age at ADHD diagnosis | Mean (SD), range |  |  |
| Use of previous ADHD medication | On stable medication |  |  |
|  | Off medication - previously used |  |  |
|  | Medication naive |  |  |
| Site | King’s College London |  |  |
|  | Southampton |  |  |
| WASI FSIQ-4 score | Mean (SD), range |  |  |

Table 2 - Baseline clinical scales

| Scale | Sample mean (SD), Median (p25,p75) or N (%) | Real eTNS  N=xx | Sham eTNS  N=xx |
| --- | --- | --- | --- |
| Parent rated ADHD-RS score |  |  |  |
| Child-rated Strength and Difficulties Questionnaire (SDQ) Hyperactivity scale subscore |  |  |  |
| Teacher-rated severity of ADHD symptoms as assessed by the Conners Teacher Rating Scale |  |  |  |
| Teacher rated ADHD-RS total score |  |  |  |
| Parent rated Irritability questionnaire total score (ARI-P) |  |  |  |
| Child rated Irritability questionnaire total score (ARI-S) |  |  |  |
| Self-rated Mind Excessive Wandering Questionnaire (MEWS) total score |  |  |  |
| Revised child and adolescent depression scale rated by children (RCADS-25) |  |  |  |
| Revised child and adolescent depression scale rated by parents (RCADS 25-P) |  |  |  |
| Columbia Suicide Severity Scale – Suicidal Ideation Score (child rated) |  |  |  |
| Cognitive performance - Vigilance Task (Omission errors %) |  |  |  |
| Cognitive performance - Vigilance Task (Commission errors %) |  |  |  |
| Parent-reported Sleep Disturbance Scale for Children total score (SDSC) |  |  |  |
| Objective hyperactivity (at 4 weeks only). This will be measured as a composite score of intensity of movement and frequency of movement. |  |  |  |
| Pupil diameter |  |  |  |
| Side effects score (rated by child) |  |  |  |
| Side effects score (rated by parent) |  |  |  |
| Weight (kg) |  |  |  |
| Pulse (bpm) |  |  |  |

## Primary outcome

Table 3 – Primary outcome results

|  | **Unadjusted mean** | |  |  |
| --- | --- | --- | --- | --- |
| **Outcome** | **Real eTNS** | **Sham eTNS** | **Adjusted Mean Difference (SE)** | **p-value (95% CI)** |
| Total ADHD score at 4 weeks |  |  |  |  |

## 9.3 Intercurrent events

Table 4 - Intercurrent events

|  | **Number of events (%)** | |  |  |
| --- | --- | --- | --- | --- |
| **Event** | **Real eTNS** | **Sham eTNS** | Hazard Ratio (SE) | p-value (95% CI) |
| Discontinuation of intervention |  |  |  |  |

## 9.4 Secondary outcomes

Table 5 - Secondary outcome results

|  | **Unadjusted,**  **Mean (SD)** | |  |  |  |
| --- | --- | --- | --- | --- | --- |
| **Outcome** | **Real eTNS** | **Sham eTNS** | **Adjusted Mean Difference (SE)** | **p-value (95% CI)** | **Cohen’s d (if applicable) (95% CI)** |
| Outcome:^*^ |  |  |  |  |  |
| 4 weeks | *n*= XX | *n*= XX |  |  |  |
| 6 months | *n*= XX | *n*= XX |  |  |  |

## Compliance

Table 6 - compliance/adherence to randomised intervention

| **Adherence to randomised intervention** | **Real eTNS**  **N (%)** | **Sham eTNS**  **N (%)** | **Total**  **N (%)** |
| --- | --- | --- | --- |
| Complied to treatment | 0/0 (%) | 0/0 (%) | 0/0 (%) |
| Completed treatment to 4 weeks | 0/0 (%) | 0/0 (%) | 0/0 (%) |
| **Withdrawn from treatment** | 0/0 (%) | 0/0 (%) | 0/0 (%) |
| *Reasons* |  |  |  |
| Child/guardian no longer wishes to have further data collected | 0/0 (%) | 0/0 (%) | 0/0 (%) |
| Unable to locate / contact child/guardian | 0/0 (%) | 0/0 (%) | 0/0 (%) |
| Child/guardian no longer able to travel to centre | 0/0 (%) | 0/0 (%) | 0/0 (%) |
| Death of child | 0/0 (%) | 0/0 (%) | 0/0 (%) |
| Death of guardian | 0/0 (%) | 0/0 (%) | 0/0 (%) |
| Other adverse event | 0/0 (%) | 0/0 (%) | 0/0 (%) |

## 9.6 Safety/Adverse events Reporting

NSAE = Non Serious Adverse Event SAE = Serious Adverse Event. NSADE = Non serious adverse device effect, SADE = Serious adverse device effect, NSAR = Non Serious Adverse Reaction, SAR = Serious Adverse Reaction

Table 7 - Number of adverse events by timepoint (cumulative)

|  | **Real eTNS** | | **Sham eTNS** | |
| --- | --- | --- | --- | --- |
| **Timepoint** | **NSAEs (NADEs)** | **SAEs (SADES)** | **NSAEs (NADEs)** | **SAEs (SADEs)** |
| 1 week | *n*= XX (n=XX) | *n*= XX (n=XX) | *n*= XX (n=XX) | *n*= XX (n=XX) |
| 2 weeks | *n*= XX (n=XX) | *n*= XX (n=XX) | *n*= XX (n=XX) | *n*= XX (n=XX) |
| 3 weeks | *n*= XX (n=XX) | *n*= XX (n=XX) | *n*= XX (n=XX) | *n*= XX (n=XX) |
| 4 weeks | *n*= XX (n=XX) | *n*= XX (n=XX) | *n*= XX (n=XX) | *n*= XX (n=XX) |
| 6 months | *n*= XX (n=XX) | *n*= XX (n=XX) | *n*= XX (n=XX) | *n*= XX (n=XX) |

Table 8 - All adverse events from baseline to 4 weeks and from baseline to 6 months follow up (separate tables will be created)

“n participants” is number of unique participants that has experienced at least one of those type of adverse events.

|  | **Real eTNS** | | **Sham eTNS** | | **Total** | |
| --- | --- | --- | --- | --- | --- | --- |
| **Type of AE** | **Number of NSAEs (n participants, %)** | **Number of SAEs (n participants, %)** | **Number of NSAEs (n participants, %)** | **Number of SAEs (n participants, %)** | **Number of NSAEs (n participants, %)** | **Number of SAEs (n participants, %)** |
| 1. Anxiety or worrying | n= (n/n, %) | n= (n/n, %) | n= (n/n, %) | n= (n/n, %) | n= (n/n, %) | n= (n/n, %) |
| 2. Tearfulness, sadness, or depression | n= (n/n, %) | n= (n/n, %) | n= (n/n, %) | n= (n/n, %) | n= (n/n, %) | n= (n/n, %) |
| 3. Tiredness, demotivation, joylessness | n= (n/n, %) | n= (n/n, %) | n= (n/n, %) | n= (n/n, %) | n= (n/n, %) | n= (n/n, %) |
| 4. Withdrawal or less socializing (less interested in peers) | n= (n/n, %) | n= (n/n, %) | n= (n/n, %) | n= (n/n, %) | n= (n/n, %) | n= (n/n, %) |
| 5. Grumpiness and irritability | n= (n/n, %) | n= (n/n, %) | n= (n/n, %) | n= (n/n, %) | n= (n/n, %) | n= (n/n, %) |
| 6. Twitching or ticks (eye blinking, head tics) | n= (n/n, %) | n= (n/n, %) | n= (n/n, %) | n= (n/n, %) | n= (n/n, %) | n= (n/n, %) |
| 7. Scratching himself/ herself, biting nails or lips more | n= (n/n, %) | n= (n/n, %) | n= (n/n, %) | n= (n/n, %) | n= (n/n, %) | n= (n/n, %) |
| 8. Headaches | n= (n/n, %) | n= (n/n, %) | n= (n/n, %) | n= (n/n, %) | n= (n/n, %) | n= (n/n, %) |
| 9. Stomach aches | n= (n/n, %) | n= (n/n, %) | n= (n/n, %) | n= (n/n, %) | n= (n/n, %) | n= (n/n, %) |
| 10. Lack of appetite | n= (n/n, %) | n= (n/n, %) | n= (n/n, %) | n= (n/n, %) | n= (n/n, %) | n= (n/n, %) |
| 11. Problems falling asleep or sleep problems | n= (n/n, %) | n= (n/n, %) | n= (n/n, %) | n= (n/n, %) | n= (n/n, %) | n= (n/n, %) |
| 12. Frustration | n= (n/n, %) | n= (n/n, %) | n= (n/n, %) | n= (n/n, %) | n= (n/n, %) | n= (n/n, %) |
| 13. Lack of confidence | n= (n/n, %) | n= (n/n, %) | n= (n/n, %) | n= (n/n, %) | n= (n/n, %) | n= (n/n, %) |
| 14. Distractibility | n= (n/n, %) | n= (n/n, %) | n= (n/n, %) | n= (n/n, %) | n= (n/n, %) | n= (n/n, %) |
| 15. Other physiological event | n= (n/n, %) | n= (n/n, %) | n= (n/n, %) | n= (n/n, %) | n= (n/n, %) | n= (n/n, %) |
| 16. Other psychological event | n= (n/n, %) | n= (n/n, %) | n= (n/n, %) | n= (n/n, %) | n= (n/n, %) | n= (n/n, %) |
| TOTAL EVENTS | n= (n/n, %) | n= (n/n, %) | n= (n/n, %) | n= (n/n, %) | n= (n/n, %) | n= (n/n, %) |

Table 9 - Body system codes for Other physiological and psychological events from baseline to 4 weeks and from baseline to 6 months (separate tables will be created)

|  | **Real eTNS** | | **Sham eTNS** | | **Total** | |
| --- | --- | --- | --- | --- | --- | --- |
| **AE Body system code** | **Number of NSAEs (n participants, %)** | **Number of SAEs (n participants, %)** | **Number of NSAEs (n participants, %)** | **Number of SAEs (n participants, %)** | **Number of NSAEs (n participants, %)** | **Number of SAEs (n participants, %)** |
| 1. Infections and infestations | n= (n/n, %) | n= (n/n, %) | n= (n/n, %) | n= (n/n, %) | n= (n/n, %) | n= (n/n, %) |
| 2. Neoplasms benign, malignant and unspecified (incl cysts and polyps) | n= (n/n, %) | n= (n/n, %) | n= (n/n, %) | n= (n/n, %) | n= (n/n, %) | n= (n/n, %) |
| 3. Blood and lymphatic system disorders | n= (n/n, %) | n= (n/n, %) | n= (n/n, %) | n= (n/n, %) | n= (n/n, %) | n= (n/n, %) |
| 4. Immune system disorders | n= (n/n, %) | n= (n/n, %) | n= (n/n, %) | n= (n/n, %) | n= (n/n, %) | n= (n/n, %) |
| 5. Endocrine disorders | n= (n/n, %) | n= (n/n, %) | n= (n/n, %) | n= (n/n, %) | n= (n/n, %) | n= (n/n, %) |
| 6. Metabolism and nutrition disorders | n= (n/n, %) | n= (n/n, %) | n= (n/n, %) | n= (n/n, %) | n= (n/n, %) | n= (n/n, %) |
| 7. Psychiatric disorders | n= (n/n, %) | n= (n/n, %) | n= (n/n, %) | n= (n/n, %) | n= (n/n, %) | n= (n/n, %) |
| 8. Nervous system disorders | n= (n/n, %) | n= (n/n, %) | n= (n/n, %) | n= (n/n, %) | n= (n/n, %) | n= (n/n, %) |
| 9. Eye disorders | n= (n/n, %) | n= (n/n, %) | n= (n/n, %) | n= (n/n, %) | n= (n/n, %) | n= (n/n, %) |
| 10. Ear and labyrinth disorders | n= (n/n, %) | n= (n/n, %) | n= (n/n, %) | n= (n/n, %) | n= (n/n, %) | n= (n/n, %) |
| 11. Cardiac disorders | n= (n/n, %) | n= (n/n, %) | n= (n/n, %) | n= (n/n, %) | n= (n/n, %) | n= (n/n, %) |
| 12. Vascular disorders | n= (n/n, %) | n= (n/n, %) | n= (n/n, %) | n= (n/n, %) | n= (n/n, %) | n= (n/n, %) |
| 13. Respiratory, thoracic and mediastinal disorders | n= (n/n, %) | n= (n/n, %) | n= (n/n, %) | n= (n/n, %) | n= (n/n, %) | n= (n/n, %) |
| 14. Gastrointestinal disorders | n= (n/n, %) | n= (n/n, %) | n= (n/n, %) | n= (n/n, %) | n= (n/n, %) | n= (n/n, %) |
| 15. Hepatobiliary disorders | n= (n/n, %) | n= (n/n, %) | n= (n/n, %) | n= (n/n, %) | n= (n/n, %) | n= (n/n, %) |
| 16. Skin and subcutaneous tissue disorders | n= (n/n, %) | n= (n/n, %) | n= (n/n, %) | n= (n/n, %) | n= (n/n, %) | n= (n/n, %) |
| 17. Musculoskeletal and connective tissue disorders | n= (n/n, %) | n= (n/n, %) | n= (n/n, %) | n= (n/n, %) | n= (n/n, %) | n= (n/n, %) |
| 18. Renal and urinary disorders | n= (n/n, %) | n= (n/n, %) | n= (n/n, %) | n= (n/n, %) | n= (n/n, %) | n= (n/n, %) |
| 19. Pregnancy, puerperium and perinatal conditions | n= (n/n, %) | n= (n/n, %) | n= (n/n, %) | n= (n/n, %) | n= (n/n, %) | n= (n/n, %) |
| 20. Reproductive system and breast disorders | n= (n/n, %) | n= (n/n, %) | n= (n/n, %) | n= (n/n, %) | n= (n/n, %) | n= (n/n, %) |
| 21. Congenital, familial and genetic disorders | n= (n/n, %) | n= (n/n, %) | n= (n/n, %) | n= (n/n, %) | n= (n/n, %) | n= (n/n, %) |
| 22. General disorders and administration site conditions | n= (n/n, %) | n= (n/n, %) | n= (n/n, %) | n= (n/n, %) | n= (n/n, %) | n= (n/n, %) |
| 23. Investigations | n= (n/n, %) | n= (n/n, %) | n= (n/n, %) | n= (n/n, %) | n= (n/n, %) | n= (n/n, %) |
| 24. Injury, poisoning and procedural complications | n= (n/n, %) | n= (n/n, %) | n= (n/n, %) | n= (n/n, %) | n= (n/n, %) | n= (n/n, %) |
| 25. Surgical and medical procedures | n= (n/n, %) | n= (n/n, %) | n= (n/n, %) | n= (n/n, %) | n= (n/n, %) | n= (n/n, %) |
| 26. Social circumstances | n= (n/n, %) | n= (n/n, %) | n= (n/n, %) | n= (n/n, %) | n= (n/n, %) | n= (n/n, %) |
| 27. Product issues | n= (n/n, %) | n= (n/n, %) | n= (n/n, %) | n= (n/n, %) | n= (n/n, %) | n= (n/n, %) |
| TOTAL EVENTS | n= (n/n, %) | n= (n/n, %) | n= (n/n, %) | n= (n/n, %) | n= (n/n, %) | n= (n/n, %) |

Table 10 - All adverse device effects from baseline to 4 weeks and from baseline to 6 months up (separate tables will be created)

(Separate tables will also report additional adverse reactions if these occur).

|  | **Real eTNS** | | **Sham eTNS** | | **Total** | |
| --- | --- | --- | --- | --- | --- | --- |
| **Type of AE** | **Number of NSADEs (n participants, %)** | **Number of SADEs (n participants, %)** | **Number of NSADEs (n participants, %)** | **Number of SADEs (n participants, %)** | **Number of SADEs (n participants, %)** | **Number of NSADEs (n participants, %)** |
| 1. Anxiety or worrying | n= (n/n, %) | n= (n/n, %) | n= (n/n, %) | n= (n/n, %) | n= (n/n, %) | n= (n/n, %) |
| 2. Tearfulness, sadness, or depression | n= (n/n, %) | n= (n/n, %) | n= (n/n, %) | n= (n/n, %) | n= (n/n, %) | n= (n/n, %) |
| 3. Tiredness, demotivation, joylessness | n= (n/n, %) | n= (n/n, %) | n= (n/n, %) | n= (n/n, %) | n= (n/n, %) | n= (n/n, %) |
| 4. Withdrawal or less socializing (less interested in peers) | n= (n/n, %) | n= (n/n, %) | n= (n/n, %) | n= (n/n, %) | n= (n/n, %) | n= (n/n, %) |
| 5. Grumpiness and irritability | n= (n/n, %) | n= (n/n, %) | n= (n/n, %) | n= (n/n, %) | n= (n/n, %) | n= (n/n, %) |
| 6. Twitching or ticks (eye blinking, head tics) | n= (n/n, %) | n= (n/n, %) | n= (n/n, %) | n= (n/n, %) | n= (n/n, %) | n= (n/n, %) |
| 7. Scratching himself/ herself, biting nails or lips more | n= (n/n, %) | n= (n/n, %) | n= (n/n, %) | n= (n/n, %) | n= (n/n, %) | n= (n/n, %) |
| 8. Headaches | n= (n/n, %) | n= (n/n, %) | n= (n/n, %) | n= (n/n, %) | n= (n/n, %) | n= (n/n, %) |
| 9. Stomach aches | n= (n/n, %) | n= (n/n, %) | n= (n/n, %) | n= (n/n, %) | n= (n/n, %) | n= (n/n, %) |
| 10. Lack of appetite | n= (n/n, %) | n= (n/n, %) | n= (n/n, %) | n= (n/n, %) | n= (n/n, %) | n= (n/n, %) |
| 11. Problems falling asleep or sleep problems | n= (n/n, %) | n= (n/n, %) | n= (n/n, %) | n= (n/n, %) | n= (n/n, %) | n= (n/n, %) |
| 12. Frustration | n= (n/n, %) | n= (n/n, %) | n= (n/n, %) | n= (n/n, %) | n= (n/n, %) | n= (n/n, %) |
| 13. Lack of confidence | n= (n/n, %) | n= (n/n, %) | n= (n/n, %) | n= (n/n, %) | n= (n/n, %) | n= (n/n, %) |
| 14. Distractibility | n= (n/n, %) | n= (n/n, %) | n= (n/n, %) | n= (n/n, %) | n= (n/n, %) | n= (n/n, %) |
| 15. Other physiological event | n= (n/n, %) | n= (n/n, %) | n= (n/n, %) | n= (n/n, %) | n= (n/n, %) | n= (n/n, %) |
| 16. Other psychological event | n= (n/n, %) | n= (n/n, %) | n= (n/n, %) | n= (n/n, %) | n= (n/n, %) | n= (n/n, %) |
| TOTAL EVENTS | n= (n/n, %) | n= (n/n, %) | n= (n/n, %) | n= (n/n, %) | n= (n/n, %) | n= (n/n, %) |

Table 11 - Body system codes for Other physiological and psychological adverse device effects from baseline to 4 weeks and from baseline to 6 months (separate tables will be created)

(Separate tables will also report additional adverse reactions if these occur).

|  | **Real eTNS** | | **Sham eTNS** | | **Total** | |
| --- | --- | --- | --- | --- | --- | --- |
| **AE Body system code** | **Number of NSADEs (n participants, %)** | **Number of SADEs (n participants, %)** | **Number of NSADEs (n participants, %)** | **Number of NSADEs (n participants, %)** | **Number of SADEs (n participants, %)** | **Number of NSADEs (n participants, %)** |
| 1. Infections and infestations | n= (n/n, %) | n= (n/n, %) | n= (n/n, %) | n= (n/n, %) | n= (n/n, %) | n= (n/n, %) |
| 2. Neoplasms benign, malignant and unspecified (incl cysts and polyps) | n= (n/n, %) | n= (n/n, %) | n= (n/n, %) | n= (n/n, %) | n= (n/n, %) | n= (n/n, %) |
| 3. Blood and lymphatic system disorders | n= (n/n, %) | n= (n/n, %) | n= (n/n, %) | n= (n/n, %) | n= (n/n, %) | n= (n/n, %) |
| 4. Immune system disorders | n= (n/n, %) | n= (n/n, %) | n= (n/n, %) | n= (n/n, %) | n= (n/n, %) | n= (n/n, %) |
| 5. Endocrine disorders | n= (n/n, %) | n= (n/n, %) | n= (n/n, %) | n= (n/n, %) | n= (n/n, %) | n= (n/n, %) |
| 6. Metabolism and nutrition disorders | n= (n/n, %) | n= (n/n, %) | n= (n/n, %) | n= (n/n, %) | n= (n/n, %) | n= (n/n, %) |
| 7. Psychiatric disorders | n= (n/n, %) | n= (n/n, %) | n= (n/n, %) | n= (n/n, %) | n= (n/n, %) | n= (n/n, %) |
| 8. Nervous system disorders | n= (n/n, %) | n= (n/n, %) | n= (n/n, %) | n= (n/n, %) | n= (n/n, %) | n= (n/n, %) |
| 9. Eye disorders | n= (n/n, %) | n= (n/n, %) | n= (n/n, %) | n= (n/n, %) | n= (n/n, %) | n= (n/n, %) |
| 10. Ear and labyrinth disorders | n= (n/n, %) | n= (n/n, %) | n= (n/n, %) | n= (n/n, %) | n= (n/n, %) | n= (n/n, %) |
| 11. Cardiac disorders | n= (n/n, %) | n= (n/n, %) | n= (n/n, %) | n= (n/n, %) | n= (n/n, %) | n= (n/n, %) |
| 12. Vascular disorders | n= (n/n, %) | n= (n/n, %) | n= (n/n, %) | n= (n/n, %) | n= (n/n, %) | n= (n/n, %) |
| 13. Respiratory, thoracic and mediastinal disorders | n= (n/n, %) | n= (n/n, %) | n= (n/n, %) | n= (n/n, %) | n= (n/n, %) | n= (n/n, %) |
| 14. Gastrointestinal disorders | n= (n/n, %) | n= (n/n, %) | n= (n/n, %) | n= (n/n, %) | n= (n/n, %) | n= (n/n, %) |
| 15. Hepatobiliary disorders | n= (n/n, %) | n= (n/n, %) | n= (n/n, %) | n= (n/n, %) | n= (n/n, %) | n= (n/n, %) |
| 16. Skin and subcutaneous tissue disorders | n= (n/n, %) | n= (n/n, %) | n= (n/n, %) | n= (n/n, %) | n= (n/n, %) | n= (n/n, %) |
| 17. Musculoskeletal and connective tissue disorders | n= (n/n, %) | n= (n/n, %) | n= (n/n, %) | n= (n/n, %) | n= (n/n, %) | n= (n/n, %) |
| 18. Renal and urinary disorders | n= (n/n, %) | n= (n/n, %) | n= (n/n, %) | n= (n/n, %) | n= (n/n, %) | n= (n/n, %) |
| 19. Pregnancy, puerperium and perinatal conditions | n= (n/n, %) | n= (n/n, %) | n= (n/n, %) | n= (n/n, %) | n= (n/n, %) | n= (n/n, %) |
| 20. Reproductive system and breast disorders | n= (n/n, %) | n= (n/n, %) | n= (n/n, %) | n= (n/n, %) | n= (n/n, %) | n= (n/n, %) |
| 21. Congenital, familial and genetic disorders | n= (n/n, %) | n= (n/n, %) | n= (n/n, %) | n= (n/n, %) | n= (n/n, %) | n= (n/n, %) |
| 22. General disorders and administration site conditions | n= (n/n, %) | n= (n/n, %) | n= (n/n, %) | n= (n/n, %) | n= (n/n, %) | n= (n/n, %) |
| 23. Investigations | n= (n/n, %) | n= (n/n, %) | n= (n/n, %) | n= (n/n, %) | n= (n/n, %) | n= (n/n, %) |
| 24. Injury, poisoning and procedural complications | n= (n/n, %) | n= (n/n, %) | n= (n/n, %) | n= (n/n, %) | n= (n/n, %) | n= (n/n, %) |
| 25. Surgical and medical procedures | n= (n/n, %) | n= (n/n, %) | n= (n/n, %) | n= (n/n, %) | n= (n/n, %) | n= (n/n, %) |
| 26. Social circumstances | n= (n/n, %) | n= (n/n, %) | n= (n/n, %) | n= (n/n, %) | n= (n/n, %) | n= (n/n, %) |
| 27. Product issues | n= (n/n, %) | n= (n/n, %) | n= (n/n, %) | n= (n/n, %) | n= (n/n, %) | n= (n/n, %) |
| TOTAL EVENTS | n= (n/n, %) | n= (n/n, %) | n= (n/n, %) | n= (n/n, %) | n= (n/n, %) | n= (n/n, %) |

Figure 2 – example dotplot of incidence of adverse events and relative risk


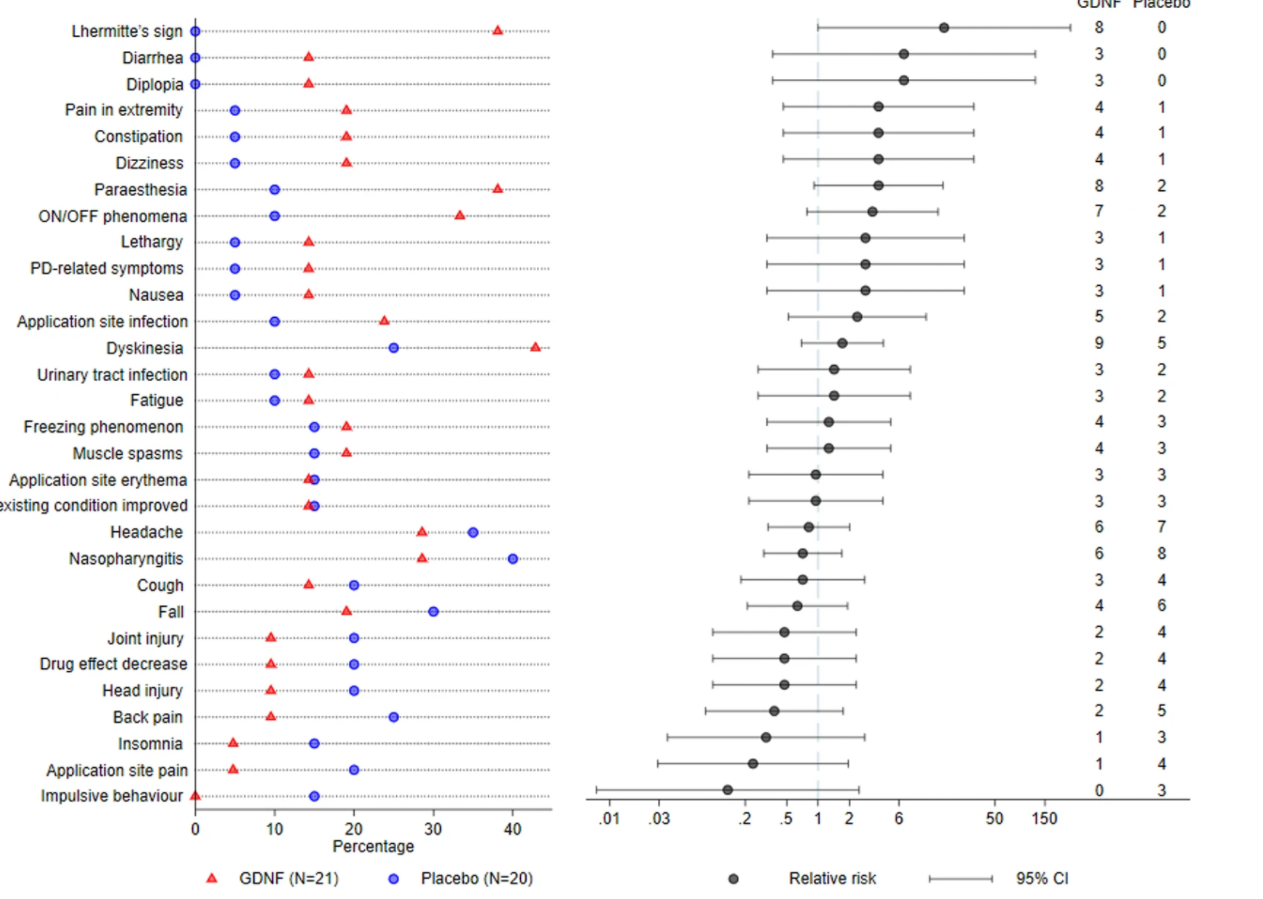


## 9.7 Unblinding events

Table 12 – participant unblinding events

| **Unblinding events** | **Real eTNS** | **Sham eTNS** |
| --- | --- | --- |
| Accidental unblinding (n, %) | n, % | n, % |
| Emergency unblinding (n,%) | n, % | n, % |

# 10. REFERENCE LIST

1. EMA. European Medicines Agency. 2018 [cited 2023 Feb 28]. ICH E9 statistical principles for clinical trials - Scientific guideline. Available from: https://www.ema.europa.eu/en/ich-e9-statistical-principles-clinical-trials-scientific-guideline

2. Schulz KF, Altman DG, Moher D, the CONSORT Group. CONSORT 2010 Statement: updated guidelines for reporting parallel group randomised trials. Trials. 2010 Mar 24;11(1):32.

3. McGough JJ, Sturm A, Cowen J, Tung K, Salgari GC, Leuchter AF, et al. Double-Blind, Sham-Controlled, Pilot Study of Trigeminal Nerve Stimulation for Attention-Deficit/Hyperactivity Disorder. Journal of the American Academy of Child & Adolescent Psychiatry. 2019 Apr 1;58(4):403-411.e3.

4. Amit O, Heiberger RM, Lane PW. Graphical approaches to the analysis of safety data from clinical trials. Pharm Stat. 2008 Mar;7(1):20–35.

5. Phillips R, Cro S. AEDOT: Stata module to produce dot plot for adverse event data [Internet]. Statistical Software Components. Boston College Department of Economics; 2021 [cited 2022 Apr 6]. Available from: https://ideas.repec.org/c/boc/bocode/s458735.html

6. White IR, Thompson SG. Adjusting for partially missing baseline measurements in randomized trials. Statistics in Medicine. 2005;24(7):993–1007.

7. Li G, Taljaard M, Van den Heuvel ER, Levine MA, Cook DJ, Wells GA, et al. An introduction to multiplicity issues in clinical trials: the what, why, when and how. International Journal of Epidemiology. 2017 Apr 1;46(2):746–55.

8. White I, Carpenter J, Horton N. A mean score method for sensitivity analysis to departures from the missing at random assumption in randomised trials. STAT SINICA [Internet]. 2018 [cited 2022 Mar 16]; Available from: http://www3.stat.sinica.edu.tw/statistica/J28N4/J28N415/J28N415.html

# 11. AMENDMENTS
